# Front‐Loading Zn^2+^ via Lattice‐Breathing‐Enabled Early‐Stage Co‐Intercalation for Ultrastable Zn‐Ion Batteries

**DOI:** 10.1002/advs.76183

**Published:** 2026-09-14

**Authors:** Haitao Li, Min Bu, Songlin Li, Huili Cao, Qi Lei, Yang Wang, Limin Zhou, Haigang Liu, Shumin Yang, Zhenhua Chen, Ying Zou, Bo Sun, Wen Wen, Yong Wang, Xiangzhi Zhang, Renzhong Tai

**Affiliations:** ^1^ Shanghai Synchrotron Radiation Facility Shanghai Advanced Research Institute Chinese Academy of Sciences Shanghai China; ^2^ State Key Laboratory of Thorium Energy Shanghai Institute of Applied Physics Chinese Academy of Sciences Shanghai China

**Keywords:** aqueous batteries, co‐intercalation, lattice breathing, manganese dioxide

## Abstract

Manganese oxide cathodes for aqueous zinc‐ion batteries present a fundamental performance dichotomy, where the rapid kinetics of H^+^ insertion are offset by structural degradation through acidification and manganese dissolution, whereas the more structurally compatible Zn^2+^ insertion is hindered by sluggish diffusion and severe lattice strain. This conventional trade‐off, stemming from the sequential or competitive nature of these ion‐storage pathways, has long constrained the achievable capacity and cycling stability. Here, we report that In^3+^ incorporation into δ‐MnO_2_ creates a dynamically adaptive lattice through controlled local strain fields, activating effective transport pathways for ultrafast charge propagation. Crucially, multimodal characterization reveals an unconventional ion‐storage mechanism in which the In^3+^‐modified host enables concerted Zn^2+^/H^+^ co‐insertion initiating at the onset of discharge. This front‐loaded co‐intercalation mechanism, facilitated by the breathing framework, ensures efficient charge compensation while minimizing deleterious H^+^‐dominant processes, thereby preserving crystallographic integrity. Consequently, the In‐δ‐MnO_2_ cathode exhibits exceptional kinetics with significantly reduced ion‐migration barriers, delivering a high specific capacity of 310.6 mAh g^−1^ at 0.5 A g^−1^ and sustaining 25 000 cycles with minimal decay at 5 A g^−1^. This work establishes dynamic lattice breathing as a generalizable design principle to reconcile fast ion transport with structural reversibility.

## Graphical abstract

This study introduces In^3+^ into δ‐MnO_2_ to create a lattice breathing effect via a dynamic strain field, which establishes efficient ″ polaronic superhighways″ for charge transport. We uncover an unconventional two‐stage (Zn^2+^/H^+^ co‐insertion followed by Zn^2+^‐dominant) mechanism that enables reversible structural adjustment. This approach simultaneously suppresses Jahn–Teller distortion and significantly lowers diffusion barriers for both Zn^2+^ and H^+^, enabling ultrafast kinetics.

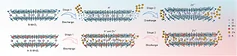

## 1 Introduction

The escalating demand for grid‐scale storage of intermittent renewable energy has propelled aqueous zinc‐ion batteries (AZIBs) to the forefront of electrochemical technologies, capitalizing on their intrinsic safety, low cost, and the high theoretical capacity (820 mAh g^−1^) of zinc metal anodes [1, 2, 3, 4, 5, 6]. Among the available cathode materials, manganese dioxide (MnO_2_) stands out for its high redox activity, natural abundance, and environmental compatibility [7, 8]. However, the practical implementation of MnO_2_ cathodes remains fundamentally limited by their poor electronic conductivity and sluggish ion‐transport kinetics, which severely restrict rate performance [9]. A more profound challenge lies in the inherent trade‐off within its storage mechanism, where H^+^ insertion provides rapid kinetics yet triggers structural acidification and manganese dissolution, while Zn^2+^ insertion, though structurally more benign, is hampered by slow diffusion and pronounced lattice strain [10, 11]. These two ion‐insertion pathways have traditionally been regarded as either sequential or competitive, imposing an irreconcilable compromise between capacity and cycling stability that persists across most transition‐metal oxide electrodes.

**FIGURE 1:**
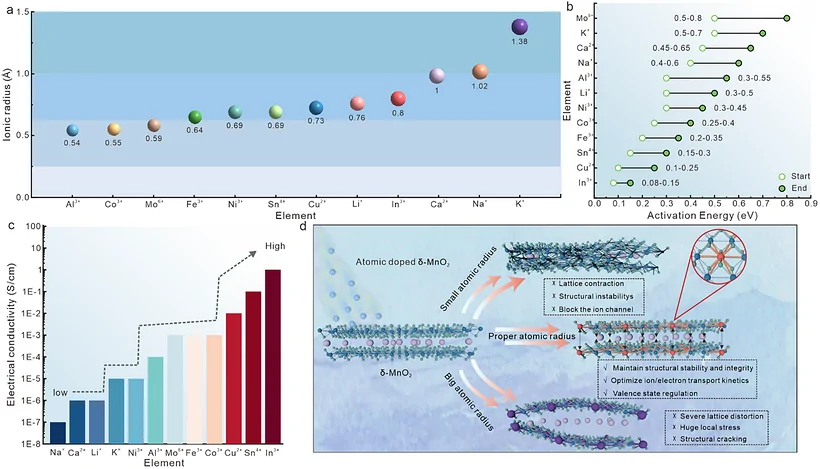
Characteristics of dopant ions. (a) Ionic radii comparison. (b) Activation energy ranges for dopant incorporation. (c) Electrical conductivity modulation by ion doping. (d) Schematic models of lattice distortion induced by ionic size mismatch.

To transcend this conventional trade‐off, we propose an alternative strategy from static electronic modification to dynamic lattice regulation via strain engineering. The core of this strategy lies in introducing specific metal ions that create a continuously adaptive local strain field at the atomic scale, thereby simultaneously tailoring charge‐transport pathways and stabilizing the host framework [12, 13]. In particular, conventional material modification strategies predominantly emphasize static electronic structure tuning, yet they frequently neglect the pivotal influence of dynamic lattice responses during ion migration, a parameter that fundamentally governs ionic transport kinetics and structural reversibility [14, 15]. We propose that surpassing this performance plateau requires active regulation of lattice dynamics through strain engineering. By incorporating a suitable dopant to create a continuously adaptive local strain field at the atomic scale, it becomes feasible to concurrently engineer efficient charge‐transport pathways and stabilize the host framework, thereby shifting the design paradigm from static electronic tuning to dynamic lattice control. Unlike long‐range structural control, this approach harnesses short‐range lattice dynamics to promote efficient electron and ion migration while preserving crystallographic reversibility [16]. Indium (In^3+^) emerges as an ideal mediator for this purpose. Its ionic radius (∼0.80 Å, six‐coordination) matches well with the Mn─O host (Figure 1a), ensuring minimal static mismatch while enabling the generation of a tunable local strain field. Moreover, In^3+^ induces a dynamic, breathing‐like distortion of metal‐oxygen bonds that maintains macroscopic symmetry yet imparts exceptional local adaptability. This strain‐mediated “lattice breathing” not only elevates carrier concentration but also establishes intermediate‐scale conduction channels conducive to rapid polaron migration (Figure 1b–d).

Herein, we demonstrate that incorporating In^3+^ into δ‐MnO_2_ creates a dynamically adaptive lattice system. Through a coordinated suite of operando synchrotron x‐ray diffraction, x‐ray absorption spectroscopy, and in situ pH monitoring, we reveal a previously unreported ion‐storage mechanism. Its most distinctive feature is the cooperative insertion of both Zn^2+^ and H^+^ from the very onset of discharge, which transitions into predominantly Zn^2+^ insertion in the later stage. Enabled by In^3+^‐induced lattice strain, this front‐loaded co‐insertion pathway establishes a well‐regulated ion‐transport network, in which dynamic lattice breathing permits reversible interlayer‐spacing adjustment while simultaneously suppressing Jahn–Teller distortions. As a result, the diffusion barriers for both Zn^2+^ and H^+^ are significantly reduced, achieving exceptional reaction kinetics without compromising structural integrity. Benefiting from this mechanism, the In‐δ‐MnO_2_ cathode exhibits remarkable electrochemical performance, delivering a high specific capacity of 310.6 mAh g^−1^ at 0.5 A g^−1^ and sustaining 25 000 cycles with minimal decay at 5 A g^−1^. Moreover, flexible full cells assembled with this cathode achieve an energy density of 473.08 Wh kg^−1^ at a power density of 270 W kg^−1^ over 100 cycles. This work not only validates dynamic lattice regulation as an effective materials‐design strategy, but more importantly, it provides a fresh crystallographic‐engineering perspective and a generalizable design principle to transcend the long‐standing trade‐off between activity and stability in intercalation‐type electrodes, offering important implications for the development of next‐generation high‐performance energy‐storage systems.

## 2 Results and Discussion

### 2.1 Characterization of Dynamic Local Lattice Distortion in In‐δ‐MnO_2_

**FIGURE 2:**
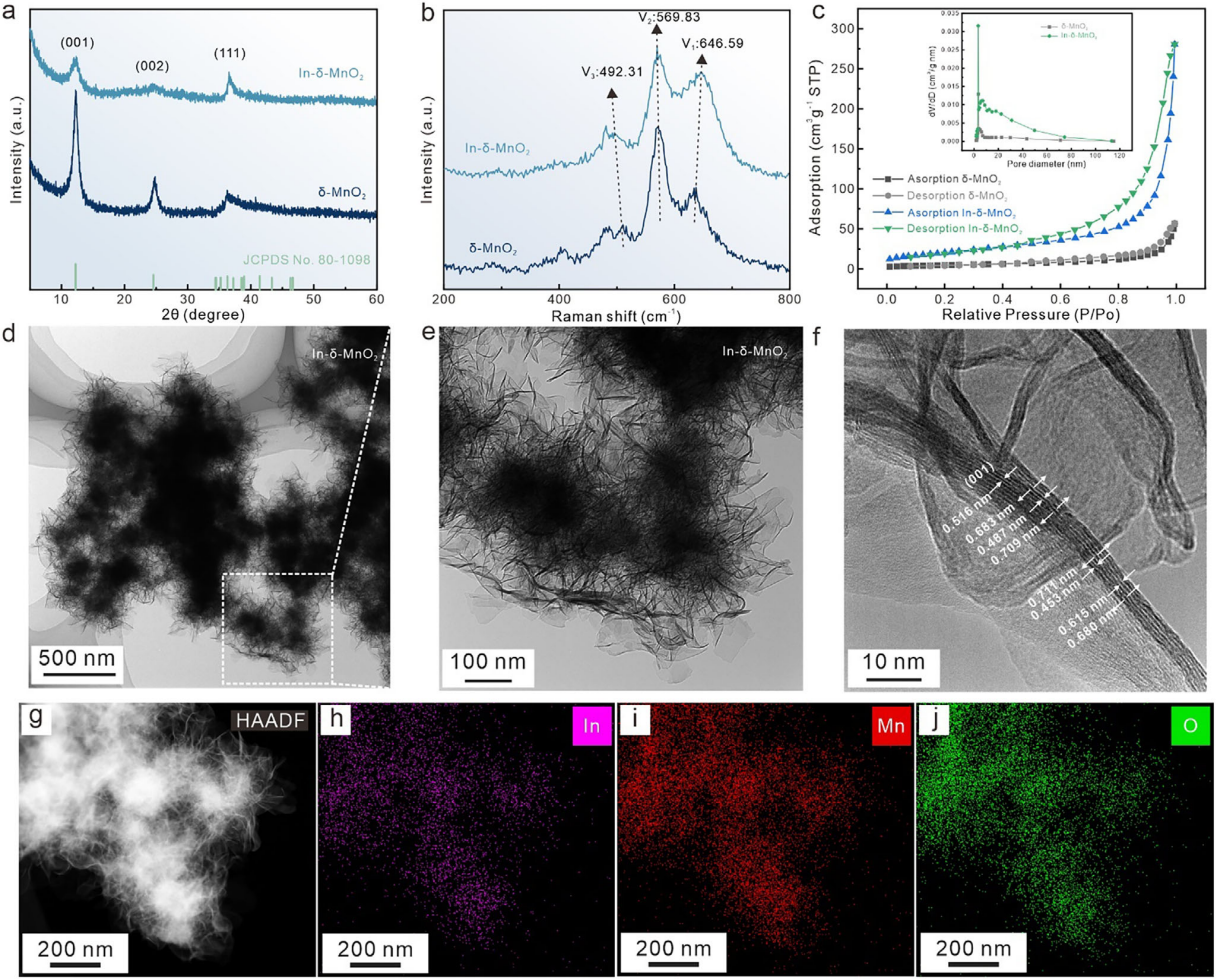
Structural and morphological characterizations. (a) XRD patterns. (b) Raman spectra. (c) N_2_ adsorption–desorption isotherms (inset: corresponding pore size distribution) of In‐δ‐MnO_2_ and δ‐MnO_2_. (d) TEM image and (e) local magnification TEM image of In‐δ‐MnO_2_. (f) HR‐TEM image. (g) HAADF image, and corresponding (h) In, (i) Mn, and (j) O element maps of In‐δ‐MnO_2_.

The integration of indium into the δ‐MnO_2_ framework via a conventional hydrothermal route yields a homogeneously modified phase, with its diffraction profile indexing unambiguously to the monoclinic C2/m space group (JCPDS 80‐1098; Figure 2a). A salient structural modulation is observed in the pronounced attenuation of (001) and (002) diffraction intensities along the c‐axis relative to the pristine phase, while their angular positions remain essentially invariant. This behavior signals the emergence of localized lattice distortions that perturb long‐range crystalline coherence without compromising the overarching structural topology. Preservation of short‐range periodicity is further affirmed by the unaltered position and intensity of the (111) reflection, indicating that the induced distortions remain spatially confined and impart anisotropic strain fields while maintaining local symmetry [17]. Raman spectroscopy provides direct evidence of In^3+^‐induced dynamic lattice anharmonicity, a key factor in polaronic state formation (Figure 2b). Contrary to the typical lattice expansion expected from a large cation, In^3+^ doping causes a blueshift of the V_1_ mode to 646.59 cm^−1^. This evidences a bond‐strengthening effect driven by enhanced octahedral symmetry. We propose that In^3+^ modifies the local electronic environment, alleviating distortion and promoting a more regular, tightly bound [MnO_6_] structure [9, 18]. In stark contrast to the V_1_ mode, the invariant V_2_ mode (octahedral base deformation) confirms that the in‐plane short‐range order is well‐preserved. This finding is consistent with the stable (111) peak in XRD, collectively ensuring the structural integrity remains intact despite the pronounced dynamic distortion. The V_3_ redshift reflects In^3+^‐driven ab‐plane compression and strengthened interlayer interaction. The concomitant strain field leads to the generation of beneficial mesoporosity, creating a percolated network of nanochannels that boost ion‐diffusion rates [9, 18]. Nitrogen physisorption isotherms delineate this In^3+^‐directed mesoarchitectural evolution (Figure 2c), exhibiting pronounced hysteresis at intermediate P/P_0_ and a monodisperse mesopore distribution centered at 3.40 nm. The fourfold augmentation in specific surface area (75.50 vs. 17.10 m^2^/g for δ‐MnO_2_) underscores the efficacy of lattice strain in driving porosity generation. This engineered pore network originates from anisotropic dilation proximal to In^3+^ sites, forging interpenetrated pathways that maximize electrolyte‐accessible interfaces. Morphological interrogation via scanning electron microscopy (SEM) captures a strain‐driven architectural metamorphosis, wherein anisotropic lattice distortion instigates spontaneous scrolling and introgressive pore formation within the lamellar matrix (Figure S1). Transmission electron microscopy (TEM) further unveils a departure from the conventional planar paradigm of MnO_2_ nanosheets (Figure 2d,e). Through deliberate lattice‐strain engineering, In^3+^ functions as a molecular‐scale architectural regulator, transmuting rigid nanosheets into an elastic, gauze‐like topography characterized by intricate curling. This reconfiguration not only alleviates inherent two‐dimensional stacking constraints but also instigates distinctive lattice‐breathing dynamics (Figure 2f). The strain landscape orchestrated by In^3+^ imposes periodic, reversible dilation and contraction upon cognately oriented (001) planes, with interlamellar spacing oscillating between 0.453 and 0.711 nm, thereby engendering spatially compartmentalized functionality. This defect‐modulated architecture integrates a three‐dimensionally bicontinuous network that operates as concurrent effective transport networks for ionic and electronic transport. Consequently, this hierarchically elastic structure directly enables the observed ultrafast charge storage kinetics by facilitating rapid mass and charge transport, while its inherent flexibility ensures durability against electrochemical cycling stress. Correlative elemental mapping via energy‐dispersive x‐ray spectroscopy (EDS), conducted at both TEM and SEM scales, provides unambiguous evidence for the homogeneous incorporation of indium species throughout the MnO_2_ host matrix (Figure 2g–j and Figure S2). This spatially uniform dopant distribution substantiates the homogeneity of the resultant lattice strain field, a structural characteristic that critically mitigates phase segregation under high‐rate ion‐intercalation conditions. Quantitative analysis by inductively coupled plasma atomic emission spectrometry (ICP‐AES) gives Mn and In mass percentages of 41.89% and 11.41%, respectively, corresponding to an In/(In+Mn) atomic ratio of approximately 11.5 at% in the bulk(Table S1). Complementary EDS spot analysis (Figure S3) yields an In/(In+Mn) atomic ratio of ∼8.3 at% (Mn: 31.0 at%, In: 2.8 at%), which is consistent with the ICP‐AES result considering the different probing depths (bulk vs. surface micro‐region). These data collectively confirm the successful and homogeneous doping of In^3+^ into the MnO_2_ lattice, establishing a stable yet locally distorted host framework.

### 2.2 Unified Electronic Structure Characterization of In‐δ‐MnO_2_

**FIGURE 3:**
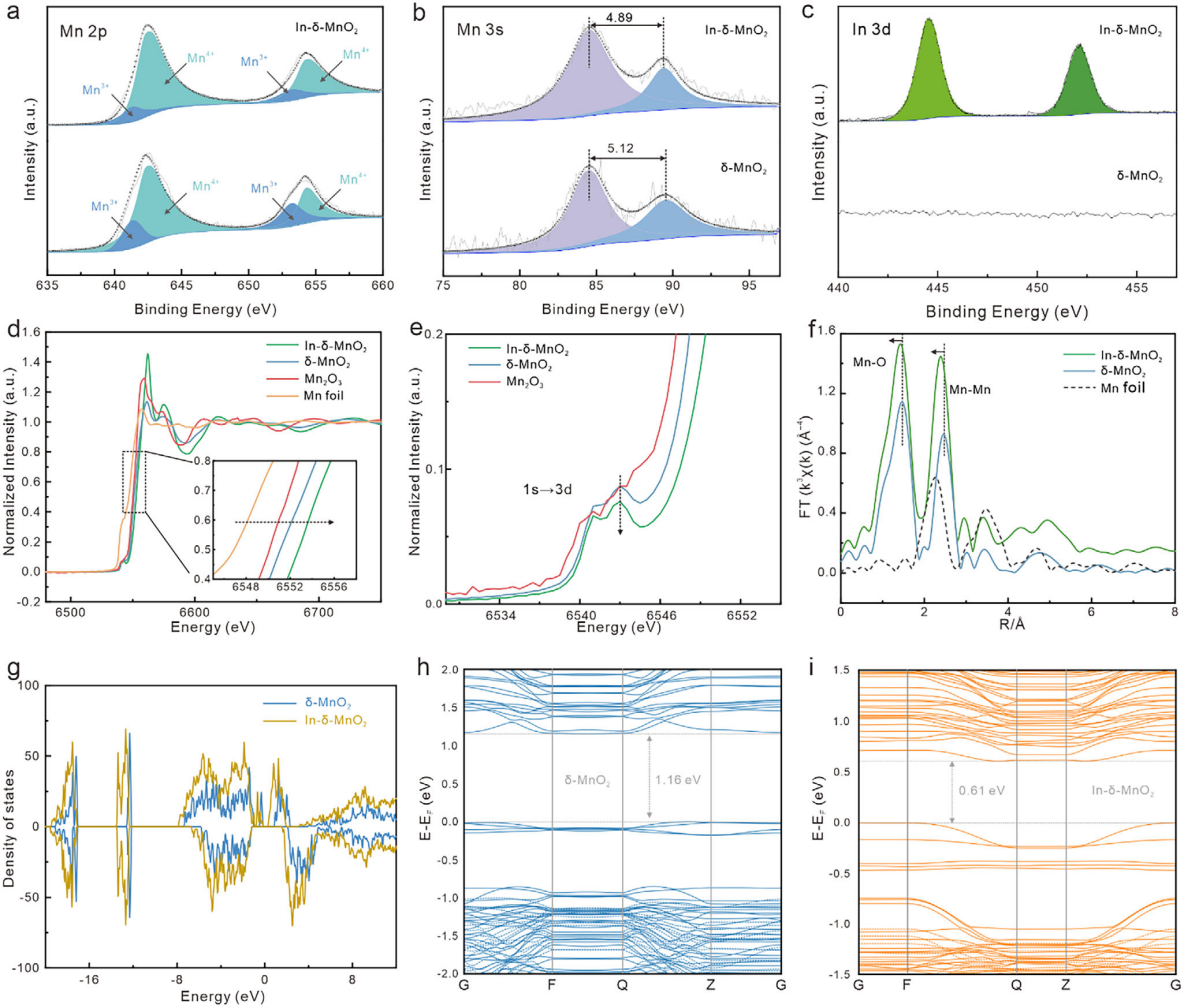
Electronic structure reconfiguration. (a) Mn 2p, (b) Mn 3s, and (c) In 3d high‐resolution XPS spectra of In‐δ‐MnO_2_ and δ‐MnO_2_. (d) Mn K‐edge XANES spectra, and corresponding (e) pre‐edge peaks. (f) Mn K‐edge FT EXAFS spectra. (g) TDOS patterns of In‐δ‐MnO_2_ and δ‐MnO_2_. Band structures of (h) δ‐MnO_2_ and (i) In‐δ‐MnO_2_.

X‐ray photoelectron spectroscopy (XPS) analysis reveals a systematic electronic reconfiguration in In‐δ‐MnO_2_ characterized by a positive binding energy shift in the Mn 2p core level and an 8.04% increase in the Mn^4+^/(Mn^3+^+Mn^4+^) ratio relative to the pristine phase (Figure 3a and Figure S4). This shift toward higher Mn oxidation states is further corroborated by the reduction of Mn 3s exchange splitting from 5.12 to 4.89 eV (Figure 3b), indicative of enhanced electron delocalization and stabilized electronic structure [19]. The observed valence redistribution, which contrasts with conventional aliovalent doping behavior, is ascribed to In^3+^‐induced compressive lattice strain that reinforces Mn─O covalent bonding. Successful indium incorporation is confirmed by the distinct In 3d spectral signature (Figure 3c), validating the proposed strain‐mediated electronic modulation mechanism. To further elucidate the associated local coordination environment, x‐ray absorption fine structure (XAFS) measurements were conducted at the Mn K‐edge. Normalized x‐ray absorption near‐edge structure spectra (XANES) exhibit a discernible positive shift in the absorption edge for In‐δ‐MnO_2_ (Figure 3d), consistent with the elevated Mn valence state identified by XPS [20]. Both materials display a pre‐edge feature corresponding to the dipole‐forbidden 1s→3d transition, enabled through 3d‐4p orbital hybridization in distorted [MnO_6_] octahedra (Figure 3e) [21]. Notably, In‐δ‐MnO_2_ demonstrates suppressed pre‐edge intensity relative to the undoped analogue, suggesting that In^3+^ incorporation mitigates Jahn–Teller distortions and enhances local symmetry. Fourier‐transformed extended x‐ray absorption fine structure (FT‐EXAFS)spectra further reveal a contraction of the Mn─O bond length from 1.51 to 1.47 Å and a concomitant shortening of the Mn─Mn distance from 2.48 to 2.45 Å (Figure 3f). This uniform bond compression originates from the compressive lattice strain generated through substitution of smaller Mn^4+^ (0.53 Å) with larger In^3+^ (0.80 Å), which strengthens orbital overlap and optimizes metal‐ligand bonding. Complementary electron paramagnetic resonance spectroscopy provides further insight into the electronic structural evolution (Figure S5). Pristine δ‐MnO_2_ exhibits pronounced spectral intensity variations across the measured field range, reflecting a heterogeneous spin environment with significant unpaired electron density characteristic of Jahn–Teller‐active Mn^3+^ species. In contrast, In‐δ‐MnO_2_ displays markedly attenuated spectral variations, indicative of a more uniform and stabilized electronic configuration. This spectral homogenization aligns with a reduced population of distortion‐prone Mn^3+^ sites and suggests the formation of localized polaronic states, both facilitated by the homogeneous compressive strain imposed by In^3+^ doping. Since Mn^3+^ is the sole origin of the Jahn–Teller distortion, the reduction in its population directly weakens the driving force for such distortion [17]. Moreover, it suppresses the disproportionation reaction of Mn^3+^ to soluble Mn^2+^, thereby mitigating the loss of active material. In the XANES pre‐edge features, the 1s→3d transition intensity of In‐δ‐MnO_2_ is markedly lower than that of the undoped counterpart, indicating a higher local symmetry of the MnO_6_ octahedra and a smaller deviation from the ideal octahedral configuration. Consequently, the population of Jahn–Teller‐active Mn^3+^ is substantially reduced. Collectively, these spectroscopic results establish that In^3+^ incorporation not only elevates and stabilizes the Mn oxidation state but also suppresses Jahn–Teller distortions through strain‐mediated symmetry preservation, thereby creating a structurally resilient host framework conducive to unconventional ion‐storage pathways.

First‐principles electronic structure calculations reveal a fundamental transformation in the electronic landscape of δ‐MnO_2_ upon indium incorporation. The total density of states (TDOS) transitions from a semiconductor‐like profile with a well‐defined bandgap in the pristine phase to one exhibiting pronounced electron density across the Fermi level in In‐δ‐MnO_2_(Figure 3g), indicative of emergent metallic character. Comparative band structure analysis further quantifies this evolution, demonstrating that In substitution reduces the fundamental bandgap from 1.16 to 0.61 eV (Figure 3h,i). This substantial band‐narrowing effectively enhances the intrinsic electronic conductivity by facilitating electron excitation and transport, thereby establishing a critical kinetic foundation for the rapid charge‐transfer processes required during high‐rate electrochemical cycling. Four‐point probe measurements confirm this enhancement, with the conductivity of In‐δ‐MnO_2_ (0.00022335 S cm^−1^) being 1.5 times that of pristine δ‐MnO_2_ (0.00014468 S cm^−1^), consistent with the bandgap narrowing (Table S2). Differential charge density analysis offers direct real‐space visualization of the associated charge redistribution (Figure S6). The maps reveal a net electron transfer from In‐coordinated oxygen sites toward the Mn─O framework, manifested as attenuated electron density around In─O bonds and intensified accumulation near Mn centers. This electronic polarization confirms In^3+^ as an effective electron donor, which strengthens Mn─O covalent interactions and stabilizes higher Mn oxidation states, in full consistency with experimental XPS and XAFS observations. The resulting charge redistribution, driven by In^3+^‐imposed compressive strain, generates an internal electric field that promotes efficient charge carrier migration while reinforcing the structural lattice. Together, these computational insights delineate a coherent electronic‐structure rationale for the superior electrode performance. The In‐induced transition toward metallic conductivity, coupled with strain‐mediated charge reorganization, collectively establishes a kinetically optimized and structurally coherent host matrix. This reconfigured electronic environment lowers ion‐migration barriers, thereby providing a fundamental theoretical basis for transcending the conventional trade‐off between insertion kinetics and structural integrity in manganese‐oxide cathodes.

### 2.3 Elucidating the Energy Storage Mechanism of the In‐δ‐MnO_2_

**FIGURE 4:**
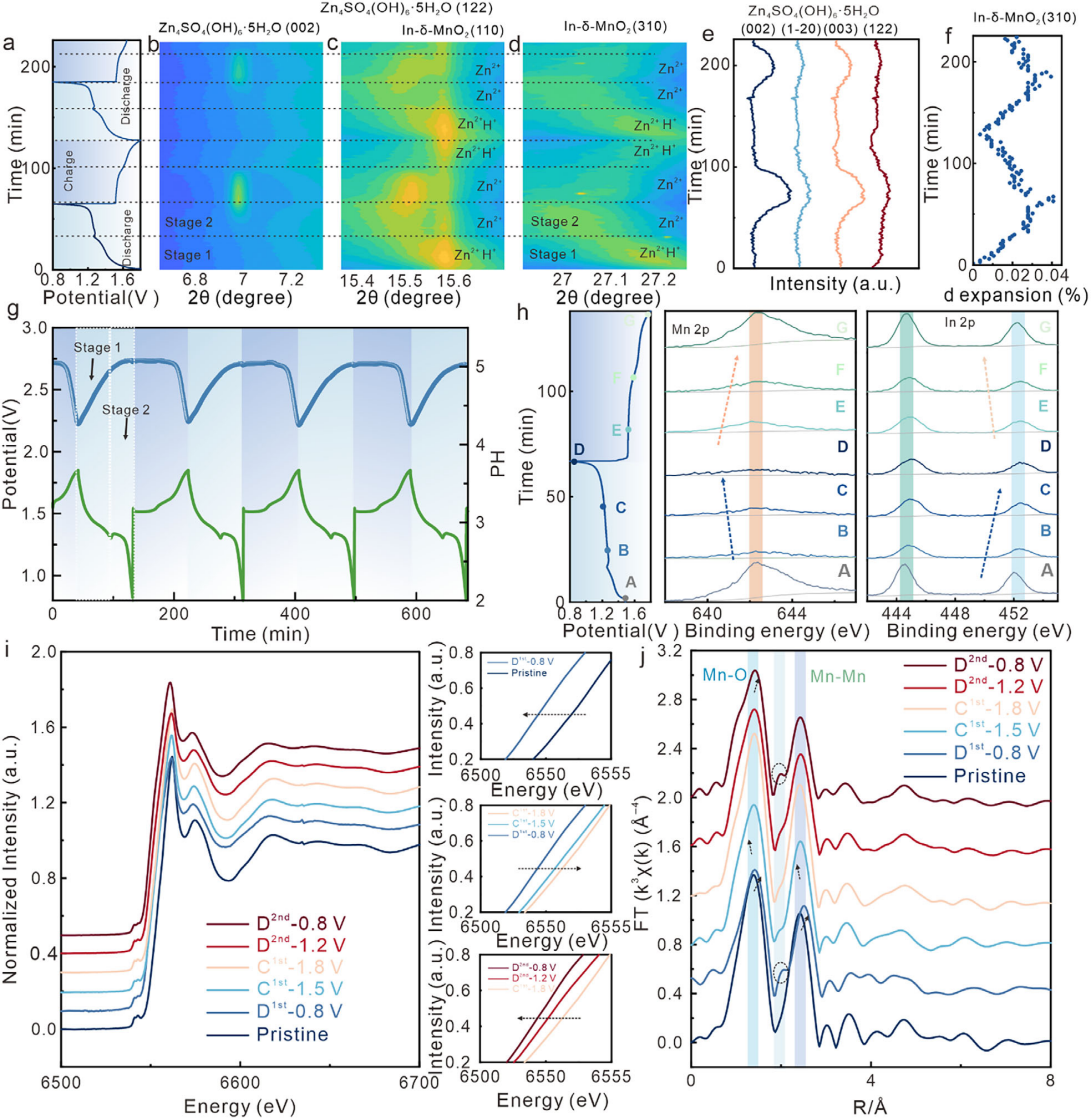
Structural evolution with electrochemical interplay in In‐δ‐MnO_2_. (a) GCD curves and (b–d) corresponding o‐SXRD patterns of typical characteristic peaks. (e) Intensity evolution of the Zn_4_SO_4_(OH)_6_·5H_2_O peaks. (f) Lattice spacing expansion in planes (310) of In‐δ‐MnO_2_. (g) In situ pH monitoring of In‐δ‐MnO_2_. (h) Ex situ XPS spectra of In‐δ‐MnO_2_ for Mn 2p and In 2p in the different state. (i) Ex situ XANES spectra at the Mn K‐edge and (j) corresponding FT‐EXAFS spectra.

To elucidate how In^3+^‐induced dynamic lattice regulation overcomes the sequential limitation of H^+^ and Zn^2+^ insertion in conventional MnO_2_ and establishes a novel cooperative intercalation mechanism from the early stage of discharge, a suite of multiscale in situ and quasi‐in situ characterization techniques was employed to systematically uncover the structural and chemical evolution of this distinct ion‐storage pathway. Operando synchrotron x‐ray diffraction (o‐SXRD, λ = 0.6887 Å) was employed over two consecutive cycles to track the evolution of characteristic diffraction peaks associated with phase transformations (Figure 4a–d). The results reveal the reversible emergence and disappearance of diffraction peaks corresponding to the zinc sulfate hydroxide hydrate (Zn_4_SO_4_(OH)_6_·5H_2_O, ZSH, JCPDS No. 39–0688) phase during discharging and charging, respectively. This evolution provides direct evidence for a concurrent H^+^/Zn^2+^ co‐insertion mechanism. The formation of ZSH is initiated by H^+^ intercalation into the In‐δ‐MnO_2_ lattice, which elevates the local OH^−^ concentration at the cathode‐electrolyte interface. The accumulated OH^−^ then reacts with Zn^2+^ and SO_4_^2−^ from the electrolyte to form ZSH [22, 23]. The anisotropic intensity variation of ZSH diffraction peaks indicates a preferential crystal growth along the c‐axis (Figure 4e). Crucially, this ZSH formation/dissolution process is highly reversible and does not impede the intrinsic ion storage of the cathode. Simultaneously, the (110) and (310) diffraction peaks of In‐δ‐MnO_2_ (at ∼15.6° and 27.2°) shift to lower angles upon discharge (Figure 4c,d), signifying an expansion of the interlayer spacing due to ion insertion. This observation diverges from the conventional two‐stage mechanism in manganese oxides, where H^+^ and Zn^2+^ insertion typically occur sequentially. The In^3+^‐induced local lattice distortion appears to effectively pre‐open the ion diffusion pathways, enabling earlier and more facile co‐insertion. The lattice expansion of the (310) plane throughout cycling follows an approximately linear trend (Figure 4f), suggesting the involvement of larger Zn^2+^ ions even in the early stage of discharge. Unlike conventional mechanisms dominated by initial H^+^ insertion, which typically induce structural acidification and manganese dissolution, the local lattice distortion engendered by In^3+^ promotes the co‐embedding of Zn^2+^ alongside H^+^ from the very beginning of discharge. A schematic comparison between the conventional proton‐dominated early insertion process and the proposed kinetically rebalanced insertion pathway is presented in Figure S7. This front‐loaded cooperative insertion effectively counteracts the detrimental interfacial acidification, thereby preserving structural integrity and mitigating manganese dissolution. The complete recovery of the peak positions after charging confirms the exceptional structural reversibility of the material. This revised mechanism is further corroborated by in situ pH monitoring (Figure 4g). The first discharge stage triggers a pronounced pH increase, confirming substantial H^+^ consumption via deep insertion. During the second discharge stage, the pH stabilizes with only minor fluctuations, indicating minimal further H^+^ involvement. The highly reversible pH evolution over full cycles underscores a robust and stable electrochemical interface, which is critical for the observed long‐term cycling stability.

Ex situ XPS analysis was performed to probe the evolution of the local chemical environment in the In‐δ‐MnO_2_ electrode during electrochemical cycling (Figure 4h). Upon discharge, the insertion of Zn^2+^ is accompanied by the reduction of Mn^4+^ to Mn^3+^ (and a small fraction of Mn^2+^) to maintain charge neutrality. This decrease in the average Mn oxidation state weakens the nuclear shielding effect, resulting in a noticeable shift of the Mn 2p peak toward lower binding energy. Correspondingly, the Zn 2p core‐level spectra reveal a progressive increase in peak intensity from the pristine state (no detectable signal) to Stage 1 and further to Stage 2 (Figure S8), and the EDS analysis confirms a similar increase in Zn mass percentage (Figure S9), directly confirming the continuous incorporation of Zn^2+^ during discharge. Concurrently, the significant incorporation of Zn^2+^ into the host structure effectively dilutes the relative atomic concentration of Mn, leading to a pronounced attenuation of the Mn 2p signal intensity. In the In 3d core‐level spectra, a shift toward higher binding energy is observed in the fully discharged state, which can be attributed to the local structural distortion induced by Zn^2+^ intercalation. This distortion likely enhances the ionic character of the In─O bond, drawing electron density away from In and thereby increasing its binding energy. Importantly, both the spectral position and intensity of the In 3d peaks fully revert to their initial states after charging, demonstrating the remarkable elasticity of the In‐δ‐MnO_2_ host framework and the highly reversible role of In sites during ion (de)intercalation.

Ex situ XAS technology was employed to investigate the evolution of short‐range structural changes in the cathode material during the charge/discharge process. The Mn K‐edge x‐ray absorption near edge structure (XANES) extracted near 6560 eV shows a profile with a pre‐edge peak during cycling (Figure 4i). It can be observed that the peak of the edge does not undergo significant changes during the cycling process. This indicates that the first coordination layer (local structure) of the Mn atom remains highly stable during the charge and discharge cycles. The bond lengths, bond angles, and covalency between Mn and the surrounding oxygen atoms have not undergone drastic and irreversible changes. This is attributed to the local lattice distortion caused by the doping of In^3+^. During the breathing‐like Zn^2+^ insertion and removal processes, this framework stabilized by In can remain basically unchanged, thereby protecting the local environment of the Mn─O polyhedron. During the discharge process, the absorption edge shifts toward lower energy. This is because when ions are embedded in the material, Mn^4+^ must acquire electrons and be reduced to Mn^3+^. The average oxidation state of Mn in the material decreases, thus causing the absorption edge to move toward lower energy [24]. The charging process, on the other hand, shifts toward higher energy. This reversible movement of the absorbing edge is direct evidence that Mn acts as the main electrochemical active center, storing and releasing energy through reversible changes in oxidation state. This is also consistent with the conclusion of XPS. By performing Fourier transformation on the Mn K‐edge during the charging and discharging processes (Figure 4j), it can be observed that, first of all, the Mn─O bond undergoes a slight elongation during the discharge process, and then returns to its original state after charging. This directly confirms the reversible valence state change of manganese. During discharge, Mn^4+^ is reduced to Mn^3+^ with a larger ionic radius and the Jahn–Teller effect, resulting in an increase in the average bond length. The oxidation process causes its reversible recovery during charging stage [25]. More importantly, the change in the Mn─Mn coordination shell reveals the influence of zinc ion insertion on the overall lattice. During discharge, the main peak of Mn─Mn shifts toward a higher R direction, reflecting the lattice expansion caused by the combined effect of Zn^2+^ insertion and the elongation of Mn─O bonds. Most crucially, during the first cycle of discharge, a new small shoulder peak appears beside the main peak of Mn─Mn. This phenomenon may be due to the new scattering path formed by the embedded Zn^2+^ and the surrounding Mn atoms (Zn─Mn bond), and the differentiation of Mn─Mn bond lengths caused by the Jahn–Teller effect of Mn^3+^. Notably, because Zn^2+^ participates in intercalation from the very beginning of discharge, it further reduces H^+^‐induced interfacial acidification and the associated generation of Mn^3+^, thereby maintaining an even lower Mn^3+^ proportion and forming a positive feedback loop for structural stability. Eventually, all these structural changes completely disappear during the charging process, fully demonstrating the excellent structural stability and reaction reversibility of the In‐MnO_2_ framework during cycling. This provides direct microscopic evidence for its excellent electrochemical cycle life.

### 2.4 Electrochemical Performance Characterization

**FIGURE 5:**
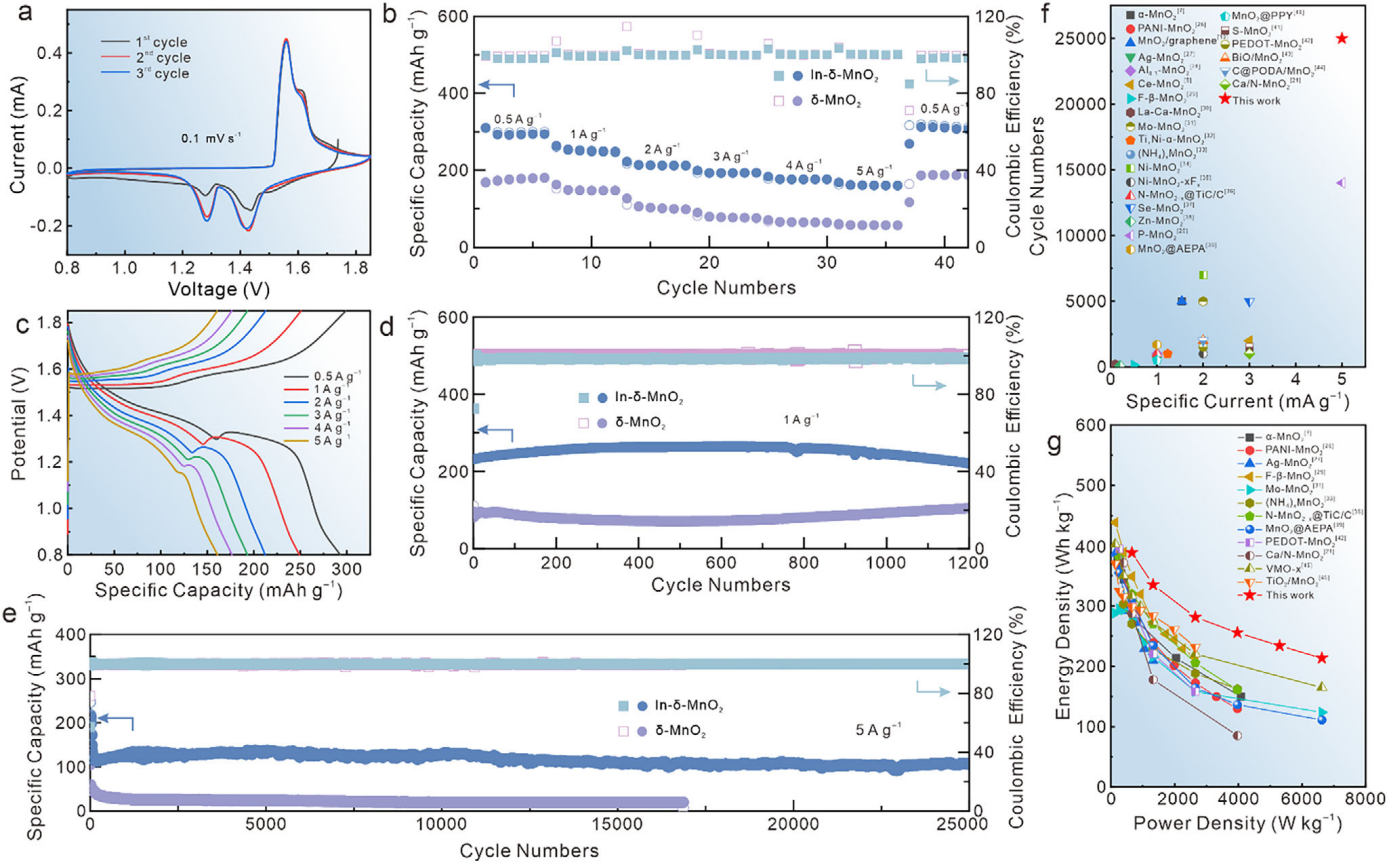
Electrochemical performance measurements. (a) CV curves of In‐δ‐MnO_2_ cathodes at 0.1 mV s^−1^. (b) The rate performance of In‐δ‐MnO_2_ and δ‐MnO_2_ at the current densities from 0.5 to 5 A g^−1^. (c) The GCD curves of In‐δ‐MnO_2_ at various current densities. (d) Cycling performance of In‐δ‐MnO_2_ and δ‐MnO_2_ at a current density of 1 A g^−1^. (e) The long‐term cycling performances of In‐δ‐MnO_2_ and δ‐MnO_2_ at 5 A g^−1^. (f) Comparison of cycle stability for the In‐δ‐MnO_2_ cathode with previously reported Mn‐based cathodes for ZIBs. (g) Comparison of energy density at different power densities.

The electrochemical behavior of the In‐δ‐MnO_2_ cathode was systematically evaluated in a coin‐type cell configuration, employing 2.0 m ZnSO_4_ with 0.2 m MnSO_4_ as the electrolyte and zinc foil as the anode. Cyclic voltammetry performed at 0.1 mV s^−1^ exhibits two distinct reduction features near 1.42 and 1.28 V, accompanied by two oxidation events at 1.56 and 1.61 V (Figure 5a). The well‐resolved redox couples, characterized by minimal voltage hysteresis, reflect highly reversible insertion‐extraction processes. This electrochemical profile aligns with the previously elucidated cooperative Zn^2+^/H^+^ intercalation mechanism, distinguishing it from the conventional stepwise insertion model and underscoring the role of In^3+^ in enabling simultaneous ion accommodation from the onset of discharge. Leveraging this kinetically optimized mechanism, the In‐δ‐MnO_2_ electrode delivers exceptional rate performance (Figure 5b). It sustains average discharge capacities of 292.94, 250.92, 212.15, 193.08, 176.55, and 160.97 mAh g^−1^ as the current density escalates from 0.5 to 5 A g^−1^. Upon reverting to 0.5 A g^−1^, the capacity recovers to 310.64 mAh g^−1^, demonstrating outstanding electrochemical reversibility. In stark contrast, the unmodified δ‐MnO_2_ cathode exhibits markedly inferior capacities under identical conditions, ranging from 177.42 to 57.5 mAh g^−1^. The corresponding galvanostatic charge–discharge profiles retain well‐defined voltage plateaus even at elevated current densities, a direct consequence of the enhanced electronic conduction and strain‐stabilized lattice imparted by In^3+^ incorporation (Figure 5c). The cycling stability and energy metrics of the In‐δ‐MnO_2_ cathode were systematically evaluated. As shown in Figure 5d, at a current density of 1 A g^−1^, the In‐δ‐MnO_2_ electrode delivers an initial discharge capacity of 231.06 mAh g^−1^. The capacity further increases during cycling, reaching a maximum of 265.09 mAh g^−1^ around the 600th cycle, likely due to a progressive activation process, and retains 95.98% of the initial capacity after 1200 cycles, demonstrating exceptional reversibility. The areal capacity values derived from Figure S10 follow the same trend and further support the superior stability of In‐δ‐MnO_2_.In contrast, the δ‐MnO_2_ cathode exhibits a markedly lower initial capacity under identical conditions. When evaluated at a high current density of 5 A g^−1^ (Figure 5e), the In‐δ‐MnO_2_ electrode exhibits exceptional long‐term cycling stability, retaining a specific capacity of 107.59 mAh g^−1^ after 25 000 cycles with near‐ideal Coulombic efficiency approaching 100%. This performance substantially surpasses that of the pristine δ‐MnO_2_ counterpart. The persistence of well‐defined voltage plateaus throughout extended cycling (Figure S11) affirms the highly reversible Zn^2+^ insertion/extraction processes enabled by the robust In‐modified framework. As summarized in Figure 5f and Table S3, the cycling stability of In‐δ‐MnO_2_ ranks among the most competitive of reported MnO_2_‐based cathodes for aqueous zinc‐ion batteries [7, 9, 10, 20, 21, 26, 27, 28, 29, 30, 31, 32, 33, 34, 35, 36, 37, 38, 39, 40, 41, 42, 43, 44]. Furthermore, the electrode achieves a notable energy density of 213.3 Wh kg^−1^ at a power density of 6625 W kg^−1^ (Figure 5g), surpassing the majority of conventional ZIB cathodes [7, 21, 26, 27, 29, 31, 33, 36, 39, 42, 45, 46]. To further evaluate the practical relevance of In‐δ‐MnO_2_, we assessed its performance under more demanding conditions, including high areal loading, a low negative‐to‐positive capacity (N/P) ratio, and a low electrolyte‐to‐capacity (E/C) ratio. As shown in Figure S12, with mass loadings ranging from 5.49 to 11.57 mg cm^−2^, the In‐δ‐MnO_2_ electrode maintains a specific capacity of 102 mAh g^−1^ after 50 cycles at the highest loading of 11.57 mg cm^−2^, corresponding to an areal capacity of approximately 1.18 mAh cm^−2^. Under a low N/P ratio of 2.09 (Figure S13), the electrode delivers a capacity of 181 mAh g^−1^ after 50 cycles, indicating excellent compatibility with lean‐anode conditions. Furthermore, with a low E/C ratio of 5 µL mg^−1^ (Figure S14), the pouch cell retains approximately 100 mAh g^−1^ after 100 cycles at 0.1 A g^−1^, demonstrating stable operation under lean‐electrolyte conditions. Collectively, these electrochemical metrics substantiate that the In^3+^‐mediated dynamic lattice regulation successfully circumvents the classical kinetics‐stability dichotomy. By fostering a synergistic ion‐storage pathway that integrates rapid H^+^‐mediated charge transfer with structurally benign Zn^2+^ accommodation, the designed cathode realizes concurrent high‐rate capability and ultralong cycle life, thereby fulfilling the unified performance paradigm set forth in this investigation.

Building on the exceptional electrochemical performance endowed by the cooperative Zn^2+^/H^+^ insertion mechanism, we further assessed the practical viability of the In‐δ‐MnO_2_ cathode through the construction of single‐layer flexible pouch cells. These devices incorporate the strain‐stabilized, mesoporous In‐δ‐MnO_2_ cathode, which preserves the dynamic lattice‐breathing characteristics detailed previously, together with a zinc‐foil anode and a mechanically robust hydrogel electrolyte. The hydrogel, formulated from cassava‐starch‐reinforced potassium polyacrylate and saturated with 1 m ZnSO_4_, exhibits high ionic conductivity while functioning as both an ion‐transport medium and a physical separator (Figures S15–S17). The assembled quasi‐solid‐state Zn||In‐δ‐MnO_2_ cell readily illuminates a commercial red light‐emitting diode, confirming operational functionality under realistic conditions (Figure S18). Electrochemical evaluation of the flexible cell demonstrates outstanding cycling stability, delivering a specific capacity of 350.43 mAh g^−1^ after 100 cycles at 0.2 A g^−1^, corresponding to 88.38% capacity retention. The associated energy density reaches 473.08 Wh kg^−1^ at a power density of 270 W kg^−1^ (Figure S19). The GCD profiles maintain well‐defined voltage plateaus with minimal polarization across cycles (Figure S20), reflecting the structural reversibility and rapid kinetics inherent to the In‐modified electrode. This behavior stems directly from the low ion‐migration barriers and capacitive‐dominated storage characteristics underpinned by the cooperative insertion mechanism. Moreover, the device displays remarkable mechanoelectrochemical stability, exhibiting open‐circuit voltage variations below 0.03 V under repeated bending to angles of 90°, 135°, and 180° (Figure S21a–d). These results collectively illustrate that the In^3+^‐driven dynamic lattice regulation, which reconciles the traditionally competing kinetics of H^+^ insertion and the structural compatibility of Zn^2+^ accommodation through early‐stage co‐intercalation, not only enhances intrinsic electrode properties but also enables the realization of durable, deformation‐tolerant energy‐storage devices. The capacity to retain high energy and power metrics under mechanical deformation underscores the practical relevance of this material‐design paradigm for next‐generation flexible electronics.

### 2.5 DFT Calculation and Kinetic Analysis

**FIGURE 6:**
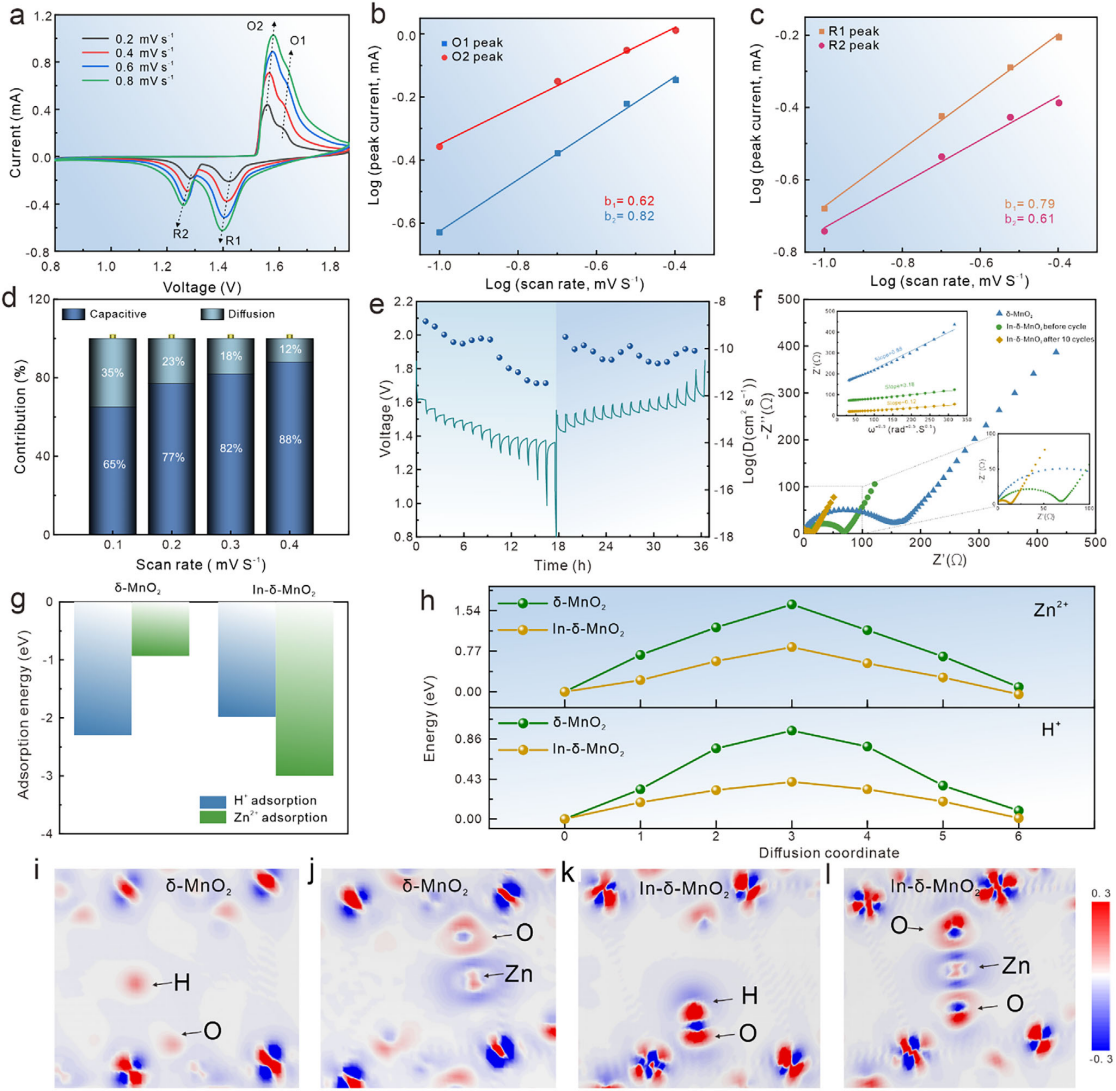
Electrochemical kinetics and theoretical mechanism insights. (a) Cyclic voltammetry curves of the In‐δ‐MnO_2_ electrode at scan rates ranging from 0.2 to 0.8 mV s^−1^. (b, c) Corresponding log(*i*) vs. log(*v*) plots derived from the anodic and cathodic peak currents, respectively. (d) Contribution ratios of capacitive and diffusion‐controlled processes at various scan rates. (e) GITT profiles and the corresponding Zn^2+^ diffusion coefficients of In‐δ‐MnO_2_. (f) Nyquist plots of In‐δ‐MnO_2_, with the inset showing the relationship between Z′ and ω^−1^/^2^ in the low‐frequency region. (g) Computed adsorption energies of Zn^2+^ and H^+^ on In‐δ‐MnO_2_ and δ‐MnO_2_. (h) Energy barriers for Zn^2+^ and H^+^ migration in δ‐MnO_2_ and In‐δ‐MnO_2_. (i–l) Differential charge density distributions of δ‐MnO_2_ and In‐δ‐MnO_2_ after Zn^2+^ or H^+^ intercalation, with red and blue isosurfaces representing charge accumulation and depletion, respectively.

The exceptional rate capability of In‐δ‐MnO_2_ prompted a systematic investigation into its electrochemical kinetics. Quantitative analysis of cyclic voltammetry profiles (Figure 6a–d) reveals that capacitive contributions dominate the charge storage process, accounting for 66%–80% of the total capacity across various scan rates. This behavior is further supported by b‐values ranging from 0.61 to 0.82, collectively indicating a surface‐controlled, rapid charge storage mechanism. This pronounced pseudocapacitive character signifies a surface‐controlled storage mechanism, enabled by the strain‐tailored architecture of In‐δ‐MnO_2_ wherein scrolled nanosheets and engineered mesoporosity collectively enhance active‐site accessibility and facilitate efficient charge delivery under high‐rate regimes. Galvanostatic intermittent titration technique (GITT) measurements reveal markedly improved Zn^2+^ diffusion kinetics, with calculated diffusion coefficients adaptively evolving within the 10^−11^ to 10^−8^ cm^2^ s^−1^ range during cycling, substantially exceeding those of most conventional intercalation‐type cathodes (Figure 6e) [47]. Complementary electrochemical impedance spectroscopy (EIS) further validates the favorable interfacial charge transfer dynamics enabled by In doping (Figure 6f). The In‐δ‐MnO_2_ electrode demonstrates an initially low charge‐transfer resistance (R_ct_) of 70.52 Ω, coupled with a near‐ideal capacitive response evidenced by a low Warburg coefficient of 0.18 Ω·s^−0.5^. More remarkably, after 10 cycles, the R_ct_ value further decreases to 15.17 Ω, indicating an activated and highly conductive interface. In stark contrast, the undoped δ‐MnO_2_ maintains a significantly higher charge‐transfer resistance throughout cycling. These results collectively underscore the critical role of In^3+^ doping in establishing rapid and stable ion/electron transport pathways, thereby ensuring superior electrochemical kinetics and interfacial stability.

DFT calculations reveal that In^3+^ doping creates a distinct host environment that selectively tunes ion adsorption energies, thereby directing the reaction pathway (Figure S22). The drastically enhanced Zn^2+^ adsorption energy (−3.00 eV vs.−0.933 eV for δ‐MnO_2_) empowers Zn^2+^ to co‐insert with H^+^ at the onset of discharge, as experimentally observed (Figure 6g). The slightly reduced H^+^ adsorption energy (−1.98 eV vs. −2.30 eV) further facilitates this cooperative insertion by preventing H^+^ from monopolizing the sites prematurely. This energetic rebalancing empowers Zn^2+^ to co‐insert with H^+^ from the very onset of discharge, thereby circumventing the conventional sequential insertion model and mitigating the deleterious interfacial acidification typically induced by dominant H^+^ uptake.

The ion migration energy barriers provide the fundamental explanation for the enhanced kinetics (Figure 6h and Figure S23). The Zn^2+^ diffusion barrier is lowered to 0.85 eV in In‐δ‐MnO_2_ from 1.66 eV in δ‐MnO_2_, and the H^+^ barrier is reduced to 0.40 eV from 0.95 eV. These substantially reduced barriers align quantitatively with the elevated diffusion coefficients from GITT and the diminished charge‐transfer resistance from EIS, confirming that the In^3+^‐modified structure alleviates kinetic limitations both at the interface and within the bulk phase. The differential charge density analysis provides direct electronic‐scale evidence for the enhanced ion‐host interactions in In‐δ‐MnO_2_ (Figure 6i–l). Upon H^+^ intercalation, the closer proximity of the H atom to the oxygen in In‐δ‐MnO_2_, coupled with the more intense red isosurface (charge accumulation) between them, indicates a stronger covalent component in the O‐H bond and a more stable chemisorption state. In contrast, the longer distance and weaker charge accumulation in δ‐MnO_2_ suggest a weaker, more physisorptive interaction. A parallel and even more pronounced effect is observed for Zn^2+^ intercalation. The tight integration of the Zn atom with the lattice oxygen in In‐δ‐MnO_2_, accompanied by profound charge accumulation in the interstitial region, signifies a markedly strengthened chemical bonding and a more complete charge‐transfer process. This signifies that Zn^2+^ is not merely physically housed within the interlayer space but forms a stable, charge‐compensated coordination with the host framework. Conversely, this integrated kinetic and theoretical analysis establishes that In^3+^‐induced lattice strain and electronic redistribution not only lower diffusion barriers and accelerate interfacial charge transfer but, more fundamentally, reconfigure the thermodynamic and kinetic landscape of ion insertion. By enabling Zn^2+^ to engage cooperatively with H^+^ from the initial discharge stage, the designed electrode transcends the classical dichotomy between proton‐mediated rapid kinetics and zinc‐ion‐imposed structural strain, thereby unifying high‐rate capability with long‐term cycling stability, a cornerstone achievement of this investigation.

## 3 Conclusion

This study successfully presents an indium‐incorporated δ‐MnO_2_ cathode that achieves outstanding overall performance in aqueous zinc‐ion batteries, a system historically limited by a fundamental compromise between proton‐mediated fast kinetics and zinc‐ion‐compatible structural stability. By introducing In^3+^ into the δ‐MnO_2_ lattice, we implement a dynamic lattice regulation strategy that reconfigures the ion storage pathway at its origin. Multimodal in situ and ex situ analyses reveal that In^3+^ incorporation instigates a continuously adaptive lattice architecture characterized by strain mediated breathing dynamics. This structural adaptability not only suppresses Jahn–Teller distortions but, decisively, unlocks an unconventional cooperative insertion mechanism. In distinct contrast to classical sequential or competitive models, Zn^2+^ and H^+^ engage in synergistic co‐insertion from the initial stage of discharge, transitioning thereafter to predominant Zn^2+^ accommodation. This front‐loaded cooperative mechanism effectively decouples the traditional trade‐off between proton‐driven kinetics and zinc ion‐induced structural deformation. The reconfigured storage pathway is energetically facilitated by substantially lowered ion migration barriers and adaptively enhanced diffusion kinetics, enabling rapid charge propagation through an interconnected three‐dimensional mesoporous network. Benefiting from this innovative design, the In‐δ‐MnO_2_ cathode achieves significantly improved electrochemical performance. It delivers a maximum specific capacity of 310.6 mAh g^−1^, exhibits outstanding rate capability, and achieves an ultralong cycling life of 25 000 cycles, compares favorably with reported values for cycling stability among manganese‐based cathodes. Furthermore, the flexible pouch cell incorporating this In‐δ‐MnO_2_ cathode achieves exceptional energy‐power characteristics, delivering an energy density of 213.3 Wh kg^−1^ at a power density of 6625 W kg^−1^ while maintaining stable open‐circuit voltage under varied testing conditions, confirms its promise for practical deployment. Fundamentally, this study establishes dynamic lattice engineering as a coherent materials design paradigm. Through precise local strain modulation, we transcend the historical compromise between insertion kinetics and structural durability in intercalation electrodes. The demonstrated confluence of mechanistic innovation and performance excellence furnishes a scalable blueprint for the development of advanced, durable energy‐storage materials extending beyond zinc‐ion systems.

## Author Contributions

**Huili Cao**: methodology. **Zhenhua Chen**: methodology. **Songlin Li**: methodology. **Shumin Yang**: methodology. **Haigang Liu**: funding acquisition. **Min Bu**: software, visualization. **Wen Wen**: methodology. **Haitao Li**: data curation, formal analysis, visualization, writing – original draft, investigation. **Yang Wang**: software. **Yong Wang**: methodology. **Xiangzhi Zhang**: conceptualization, funding acquisition, validation, investigation. **Bo Sun**: methodology. **Ying Zou**: methodology. **Renzhong Tai**: conceptualization, validation, investigation. **Qi Lei**: conceptualization, formal analysis, funding acquisition, project administration, resources, writing – review and editing, visualization, validation, supervision, data curation, investigation. **Limin Zhou**: methodology.

## Conflicts of Interest

The authors declare no conflicts of interest.

## Supporting information




**Supporting File**: advs76183‐sup‐0001‐SuppMat.pdf.

## Data Availability

The data that supports the findings of this study are available in the supplementary material of this article.
