# Supplementary material for: Front‐Loading Zn2+ via Lattice‐Breathing‐Enabled Early‐Stage Co‐Intercalation for Ultrastable Zn‐Ion Batteries
Source: Adv Sci (Weinh). 2026 Sep 14;13(58):e76183. doi: 10.1002/advs.76183 (PMC13573639; doi:10.1002/advs.76183)
Supplement: Supplementary file 1 [file ADVS-13-e76183-s001.pdf]

Supplementary information

Front-Loading Zn^{2+} via Lattice-Breathing-Enabled Early-Stage Co-Intercalation for Ultrastable Zn-Ion Batteries

Haitao Li[1,3], Min Bu[2,3], Songlin Li[1], Huili Cao[1], Qi Lei[*1], Yang Wang[2], Limin Zhou[1], Haigang Liu[1], Shumin Yang[1], Zhenhua Chen[1], Ying Zou[1], Bo Sun[1], Wen Wen[1], Yong Wang[1], Xiangzhi Zhang[*1], Renzhong Tai[*1]

[1]*Shanghai Synchrotron Radiation Facility, Shanghai Advanced Research Institute, Chinese Academy of Sciences, Shanghai 201204, China*

[2]*State Key Laboratory of Thorium Energy, Shanghai Institute of Applied Physics, Chinese Academy of Sciences, Shanghai 201800, China*

[3]*These authors contributed equally to this work.*

* Corresponding author: leiq@sari.ac.cn (Qi Lei), zhangxz@sari.ac.cn (Xiangzhi Zhang), tairz@sari.ac.cn (Renzhong Tai).

Experimental Section

Material preparation

The In-δ-MnO_2 material was synthesized via a facile one-step hydrothermal route. In a typical synthesis, 0.0125 mol of potassium permanganate ($KMnO_4$, Sinopharm Chemical Reagent, 99.5%), 0.002 mol of manganese sulfate monohydrate ($MnSO_4 \cdot H_2O$, Aladdin, 98.0%), and 0.04 mol of indium chloride tetrahydrate ($InCl_3 \cdot 4H_2O$, Sinopharm Chemical Reagent, AR) were sequentially dissolved in 30 mL of deionized water under continuous stirring for 1 hour. The resulting homogeneous solution was transferred into a 50 mL Teflon-lined stainless-steel autoclave and subjected to hydrothermal treatment at 140 °C for 10 hours, followed by natural cooling to room temperature. The solid product was collected by centrifugation, washed thoroughly with deionized water and ethanol in three successive cycles, and finally dried in a vacuum oven at 60 °C for 12 hours. For comparison, pristine δ-MnO_2 was prepared under identical conditions in the absence of $InCl_3 \cdot 4H_2O$.

Characterization Methods

The crystal structure of the samples was characterized by X-ray diffraction (XRD, Bruker D2 Phaser) using Cu Kα radiation (λ = 1.5418 Å). Raman spectroscopy was performed on a Renishaw inVia Basis system with a 532 nm laser source. Specific surface area and pore structure were evaluated through nitrogen physisorption measurements (Micromeritics ASAP 2020) based on the Brunauer-Emmett-Teller (BET) method. Morphological and elemental distribution analyses were conducted using scanning electron microscopy (SEM, Zeiss Gemini SEM 300) equipped with energy-dispersive X-ray spectroscopy (EDS), as well as transmission electron microscopy (TEM, FEI Titan Cubed Themis G2300) with EDS capability. Chemical states were investigated by X-ray photoelectron spectroscopy (XPS, Thermo Scientific K-Alpha), with the C 1s peak at 284.6 eV serving as the binding energy reference. Mn K-edge X-ray absorption spectroscopy (XAS) was conducted at the BL16U beamline of the Shanghai Synchrotron Radiation Facility (SSRF) in total fluorescence yield (TFY) mode. Data processing including background subtraction and normalization was performed using the ATHENA software package. Local pH evolution near the cathode/electrolyte interface was monitored in situ using a digital pH meter (PHSJ-4F, Shanghai Leici) with recordings taken every 30 seconds. The electronic conductivity was measured using a four-point probe method on a FOUR DIMENSIONS-280I instrument.

Electrochemical measurements

The In-δ-MnO_2 electrode was fabricated by thoroughly mixing the active material, Super P carbon, and polyvinylidene difluoride (PVDF) in a weight ratio of 7:2:1 in N-methyl-2-pyrrolidone (NMP) to form a homogeneous slurry. This slurry was uniformly coated onto a carbon cloth current collector and dried under vacuum at 60 °C for 12 h. The resulting mass loading of the active material was controlled between 1 and 2 mg cm^{-2}. CR2025-type coin cells were assembled in ambient conditions using the as-prepared In-δ-MnO_2 cathode, a 0.2 mm thick zinc foil anode, a Whatman GF/D glass fiber separator, and an aqueous electrolyte consisting of 3 M $ZnSO_4$ and 0.2 M $MnSO_4$ (approximately 90 µL per cell). Galvanostatic testing and galvanostatic intermittent titration technique (GITT) were carried out on Neware battery test systems. The GITT protocol applied a current pulse of 0.2 A g^{-1} for 5 min followed by a 60 min relaxation period. Cyclic voltammetry (CV) and electrochemical impedance spectroscopy (EIS) were performed on a CHI 600E electrochemical workstation. EIS measurements were conducted at the open-circuit potential over a frequency range of 0.01 Hz to 100 kHz with an AC amplitude of 10 mV. For ex situ characterization (XAS, XPS, and TEM), cycled electrodes were carefully extracted from disassembled cells and rinsed repeatedly with deionized water.

A mechanically robust hydrogel electrolyte was synthesized via a multistep procedure. First, 0.15 g of cassava starch was hydrothermally treated at 90 °C for 1 h in 3 mL of deionized water to obtain a viscous gel. Separately, 3.936 g of potassium hydroxide was dissolved in 15 mL of water, followed by the sequential addition of 6 g of acrylic acid, 0.0084 g of potassium persulfate (initiator), and 0.009 g of N,N'-methylene bisacrylamide (crosslinker) under continuous stirring for 40 min. The resulting potassium polyacrylate precursor was then slowly blended with the starch gel over 1 h and thermally cured at 80 °C for 3 h to form a covalently cross-linked network. The synthesized hydrogel was fully saturated with a 3 M $ZnSO_4$ electrolyte solution. Flexible quasi-solid-state In-δ-MnO_2||Zn cells were constructed by assembling the same electrode configuration as the liquid electrolyte cells,

replacing the liquid electrolyte and separator with the hydrogel electrolyte, and finally vacuum-sealing the package. All specific capacity and energy density values reported in this work are calculated based on the mass of the cathode active material (In-δ-MnO_2).

Specifically, the energy density was calculated using the following formula:

$$E = \frac{C \times V}{m_{\text{cathode}}}$$

V is the average discharge voltage (V), obtained by integrating the galvanostatic charge-discharge curve, C is the discharge capacity (mAh), $m_{cathode}$ is the mass of the active cathode material (In-δ-MnO_2) in the electrode.

Operando synchrotron characterization

Operando structural evolution during electrochemical cycling was investigated with high temporal resolution using synchrotron hard X-rays (λ = 0.6887 Å, 18 keV) at the BL14B beamline of the Shanghai Synchrotron Radiation Facility (SSRF). A custom-designed CR2025 coin cell was fabricated for X-ray transmission measurements, featuring a 2.0 mm diameter X-ray window aligned by laser collimation and sealed with a 25 μm polyimide film (Kapton® HN). The cell was assembled with an In-δ-MnO_2 working electrode, glass fiber separator, 100 μL of 2 M $ZnSO_4$ + 0.2 M $MnSO_4$ aqueous electrolyte, and a zinc foil counter electrode with a 1 mm central aperture. Galvanostatic cycling at 0.2 A g^{-1} was maintained using a LAND CT2001A system, with the ambient temperature controlled at 25 °C. Two-dimensional diffraction patterns were continuously recorded by a PILATUS3S 2M detector and subsequently integrated using the Fit2D software package for detailed phase analysis.

Calculation

All density functional theory (DFT) calculations were carried out using the CASTEP module implemented in the Materials Studio package[1]. The exchange-correlation interactions were treated within the framework of the generalized gradient approximation (GGA) using the Perdew-Burke-Ernzerhof (PBE) functional[2]. To properly describe the strong on-site Coulomb interactions of Mn 3d electrons, the DFT+U method was employed with an effective U value of 3.9 eV[3]. The long-range van der Waals interactions were corrected using the semi-empirical DFT-D3 scheme[4].

A plane-wave basis set with a kinetic energy cutoff of 500 eV was used for all calculations. The Brillouin zone was sampled using a 2×2×1 Monkhorst-Pack k-point mesh. The total energy convergence threshold was 1×10^{-6} eV/atom, and the maximum force on each atom was less than 0.03 eV/Å[3]. A 2×3×1 supercell was constructed to reduce periodic image interactions and ensure accurate structural representation. To eliminate artificial interactions between adjacent slabs, a vacuum layer of 15 Å was introduced along the z-direction[5]. The diffusion energy barriers for Zn^{2+}/H^+ in MnO_2 and In-MnO_2 were calculated using the complete LST/QST method.

Figure S1. SEM images of In-δ-MnO_2.

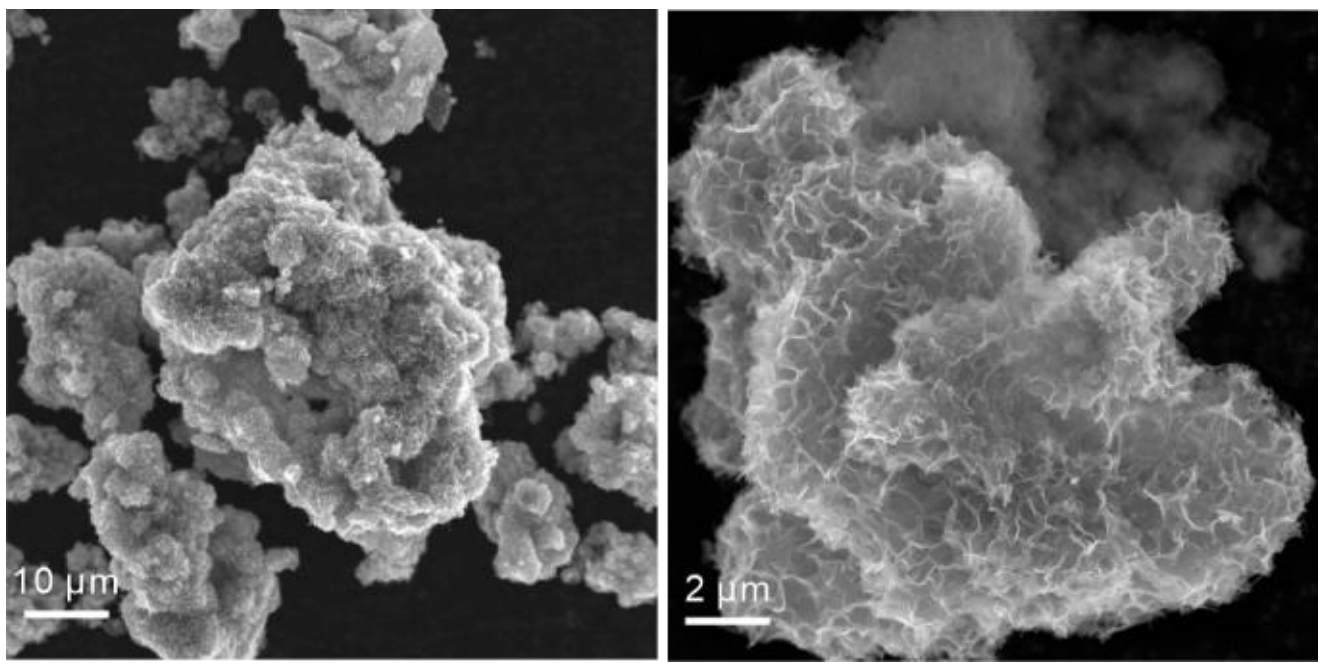

Figure S2. (a) SEM image and (b-d) corresponding elemental maps (O, Mn, In) of In-δ-MnO_2.

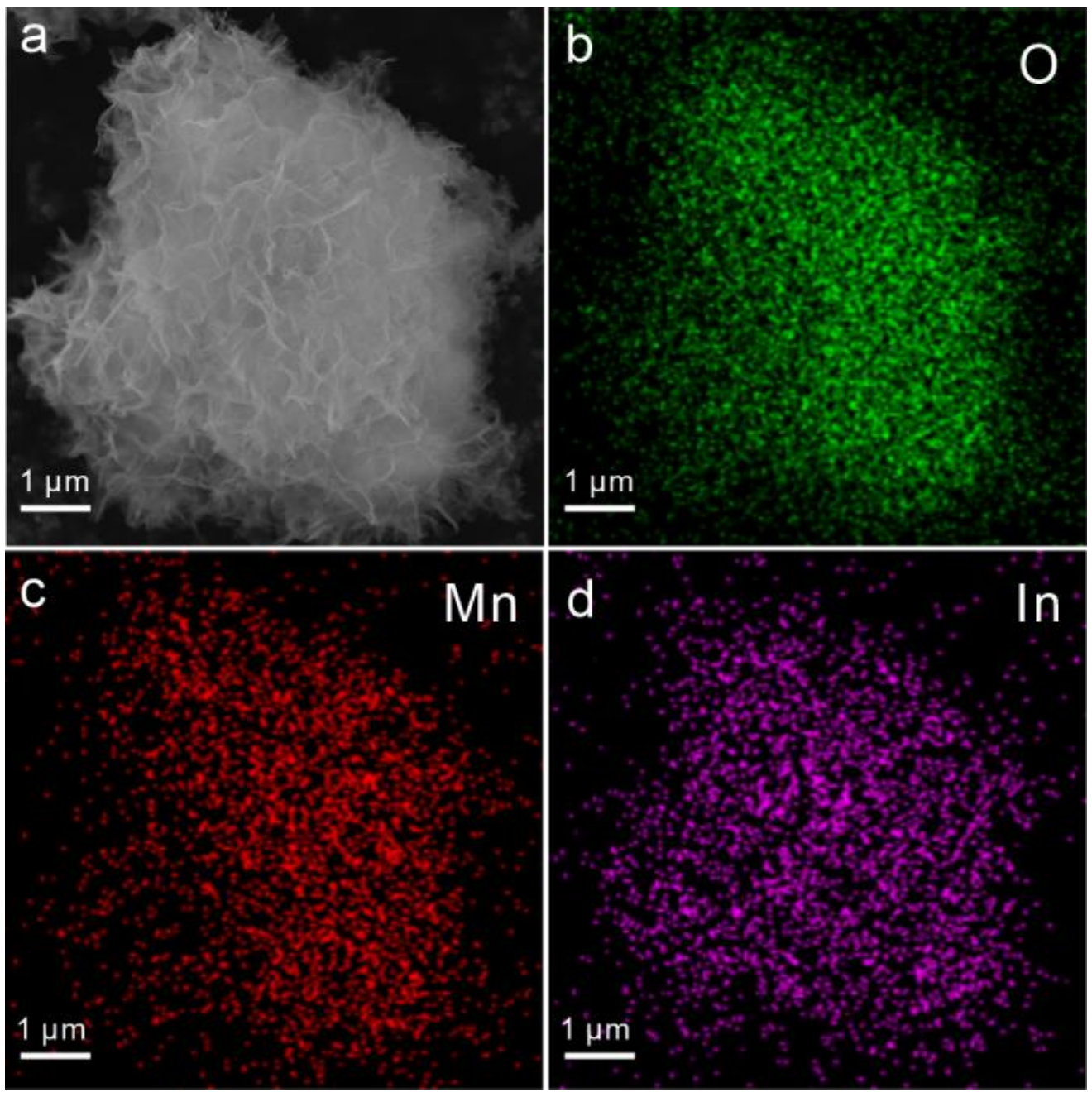

Figure S3. SEM image and the corresponding EDS spectra, with the atomic percentages of surface elements.

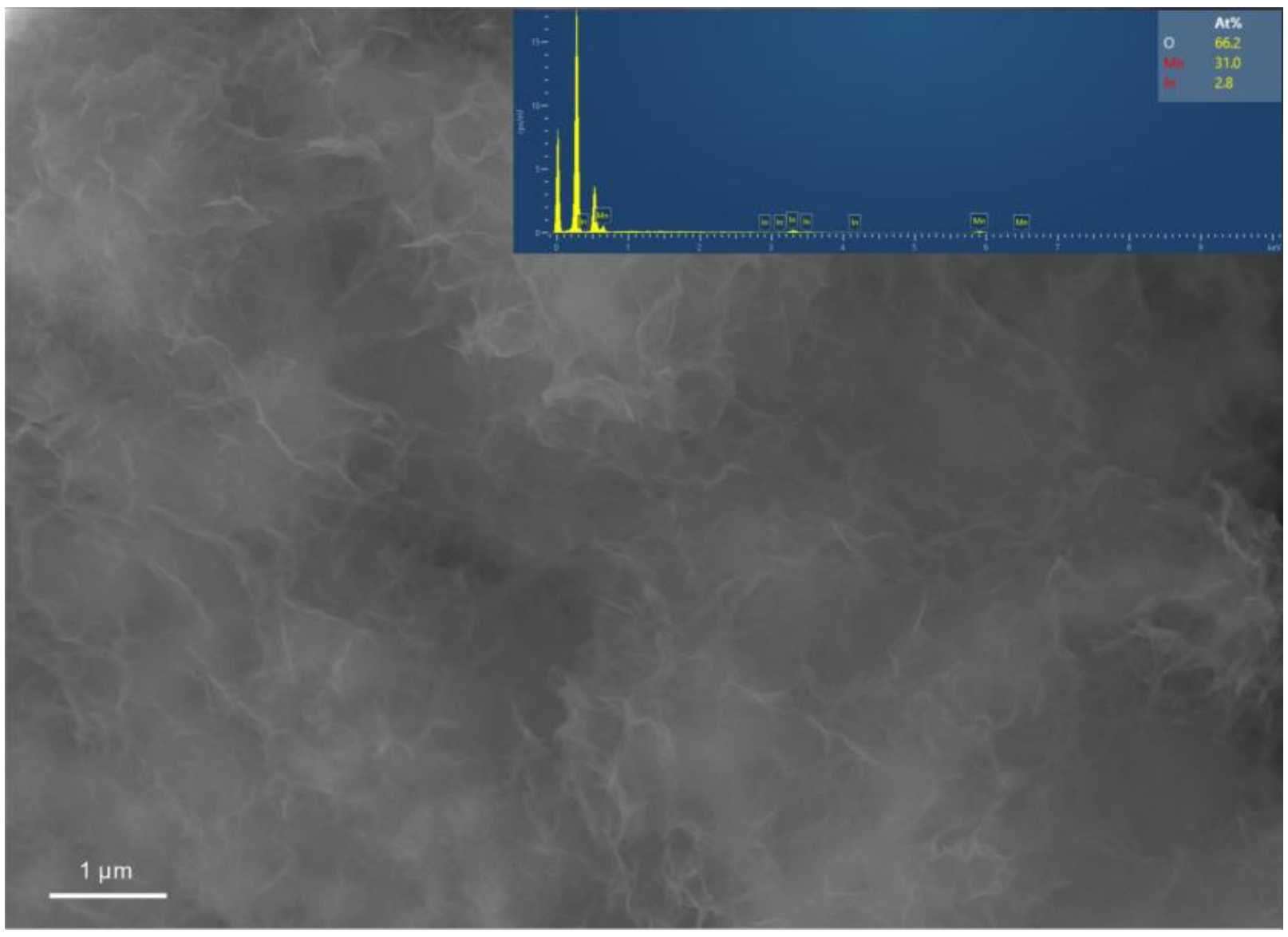

Figure S4. The full XPS spectrum of In-δ-MnO_2.

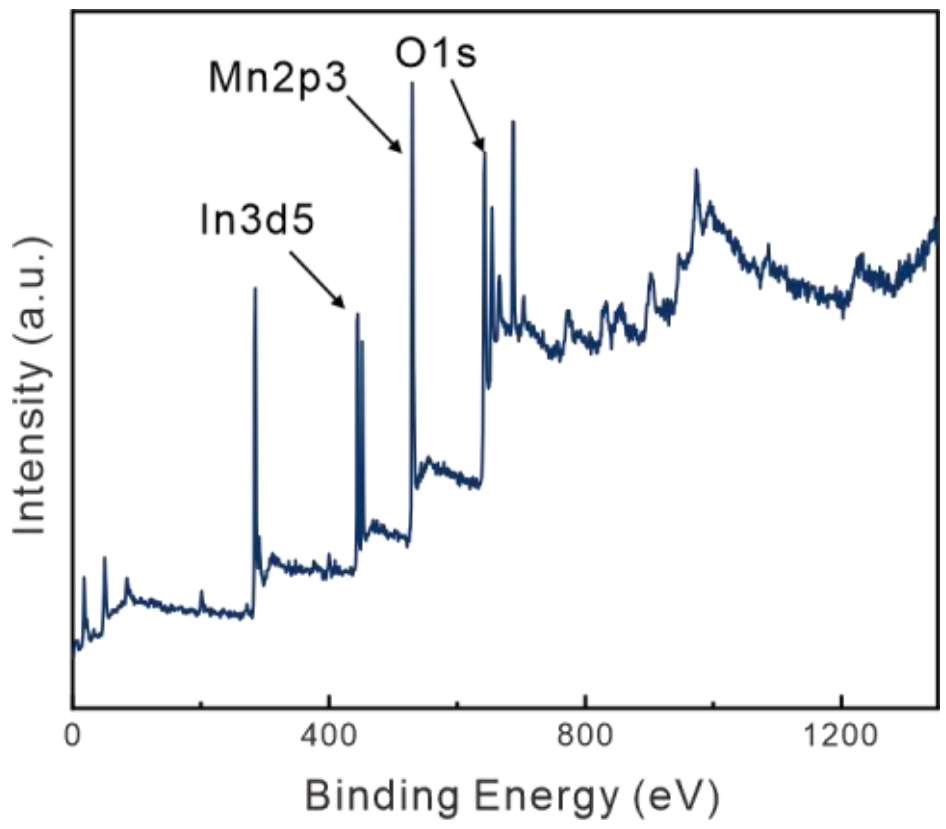

Figure S5. Comparative EPR spectra of In-δ-MnO_2 and δ-MnO_2.

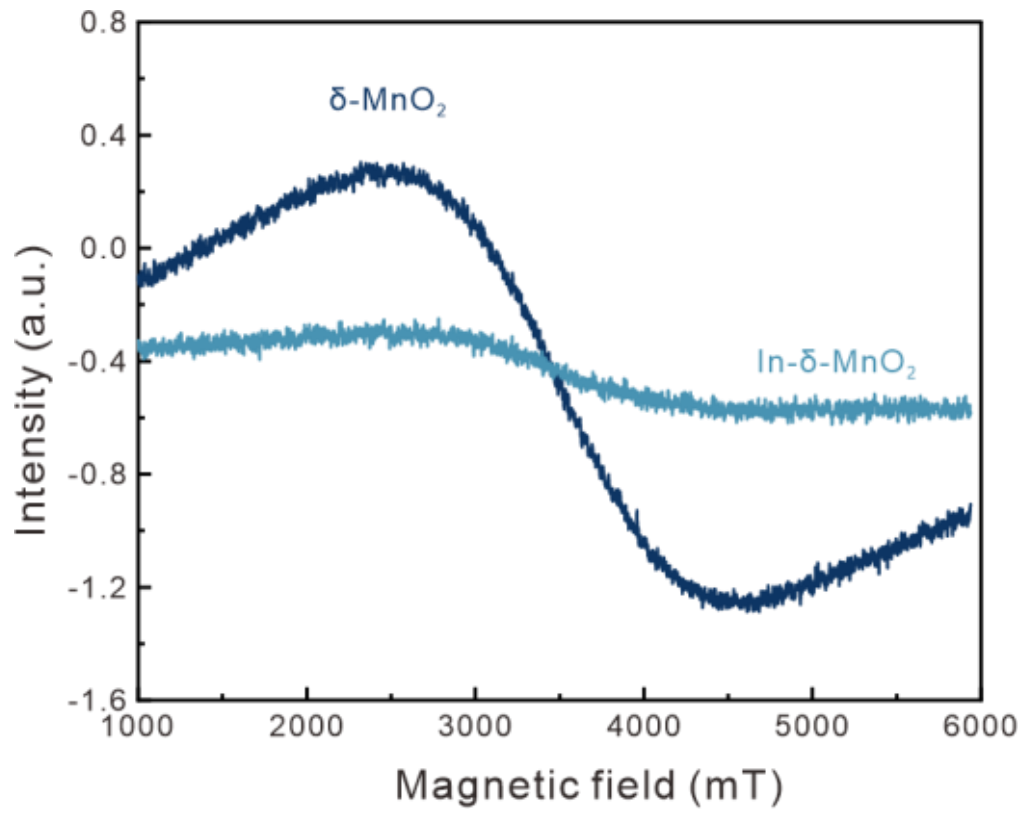

Figure S6. Differential charge density analysis of (a) δ-MnO_2 and (b) In-δ-MnO_2, where the blue area represents electron depletion and the red area represents electron accumulation.

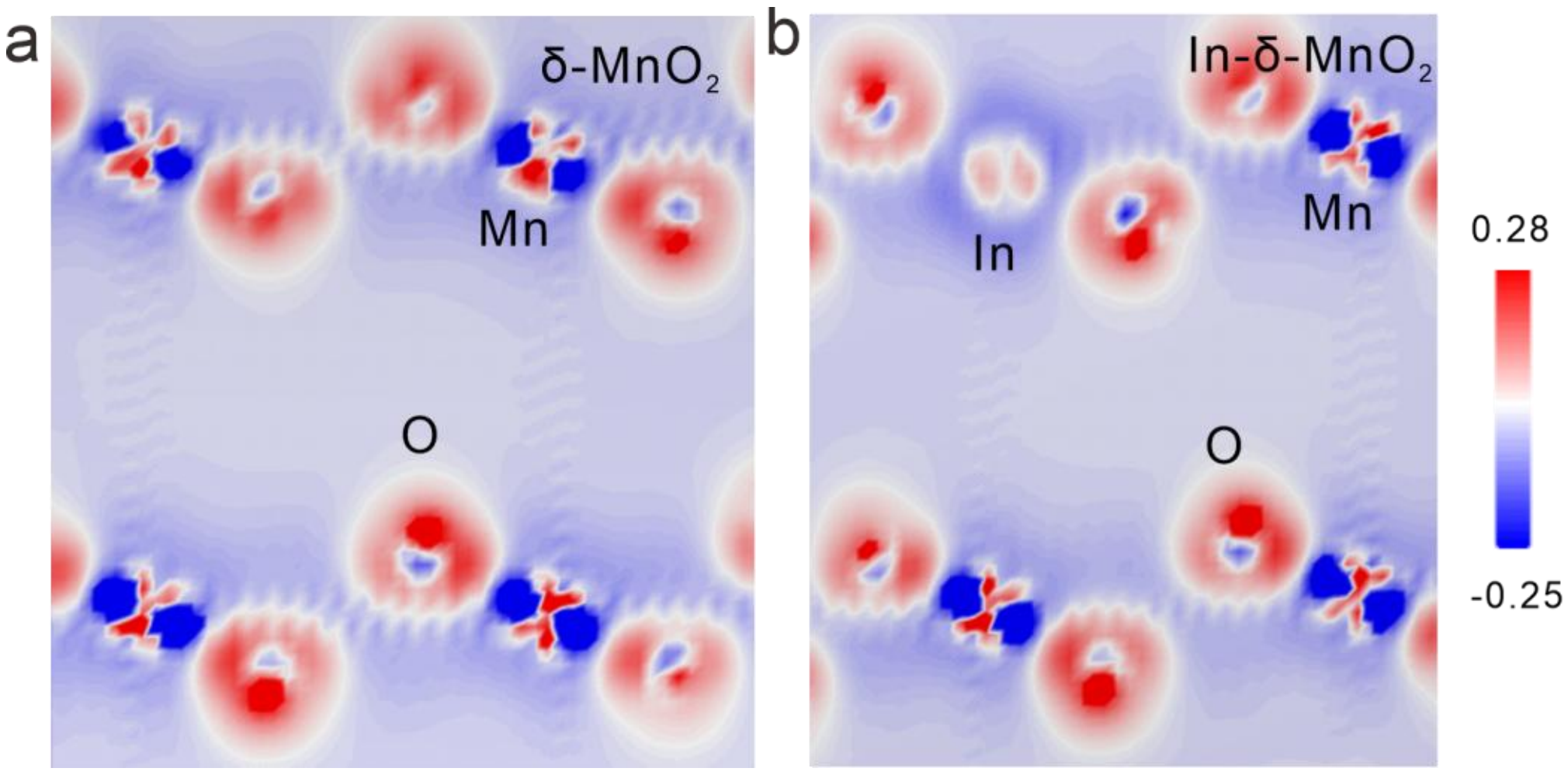

Figure S7. Schematic comparison between the conventional proton-dominated early insertion process in δ-MnO_2 and the proposed kinetically rebalanced insertion pathway in In-δ-MnO_2.

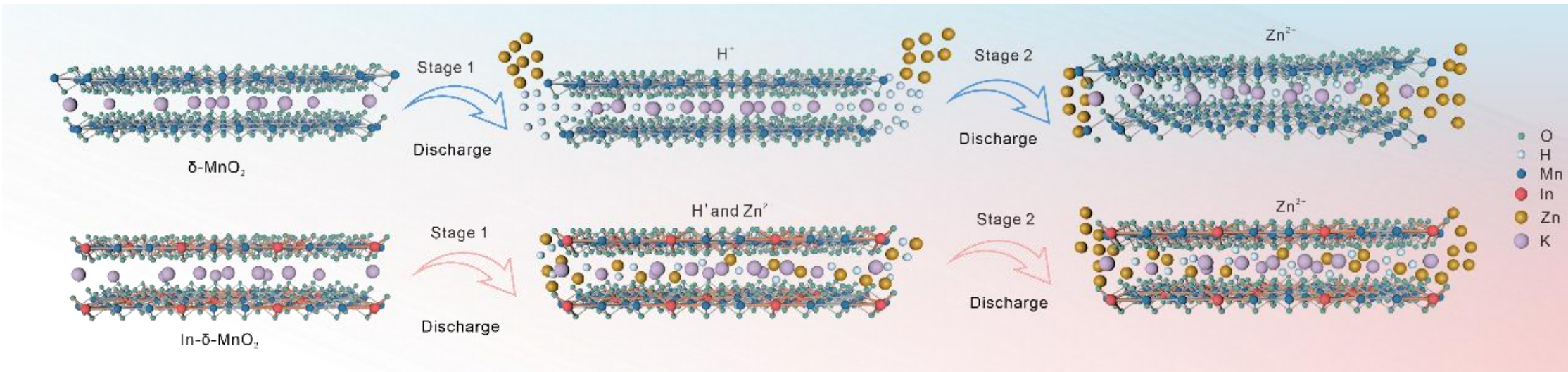

Figure S8. XPS spectra of pristine electrode and the electrode at the various stages.

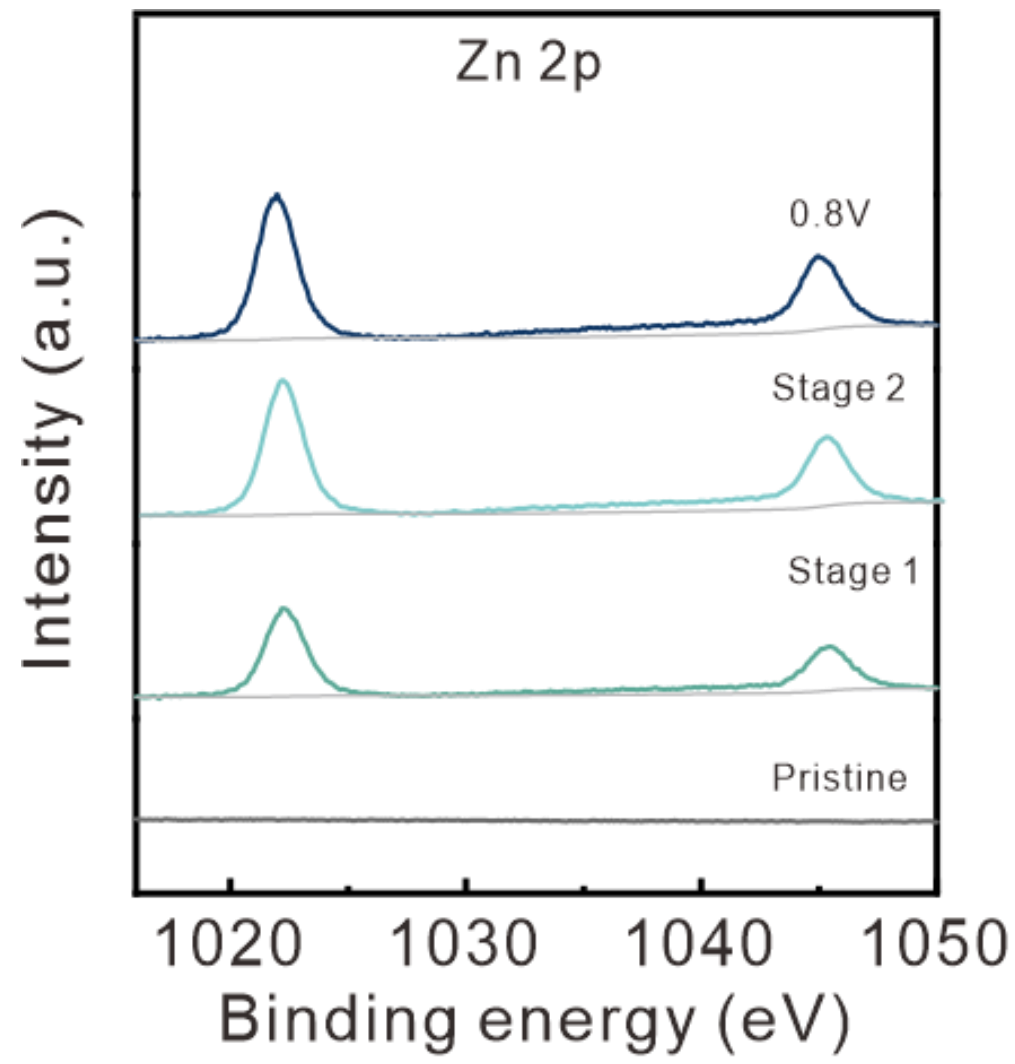

Figure S9. (a, c, e) EDS elemental maps of Mn, In, and Zn, respectively, demonstrating the homogeneous distribution of dopants and intercalated Zn^{2+}. (b, d, f) EDS spectra acquired from different regions, showing the corresponding variations in the mass percentage of surface element content.

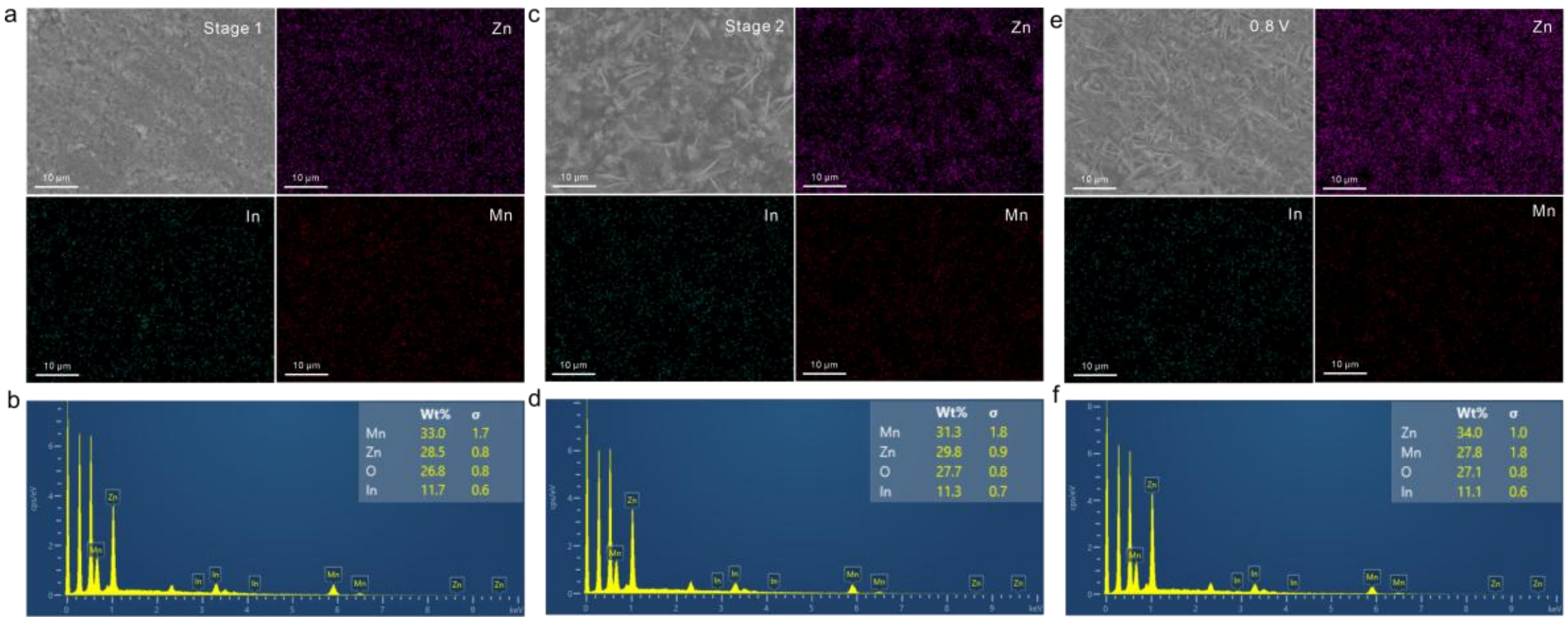

Figure S10. Areal capacity of δ-MnO_2 and In-δ-MnO_2.

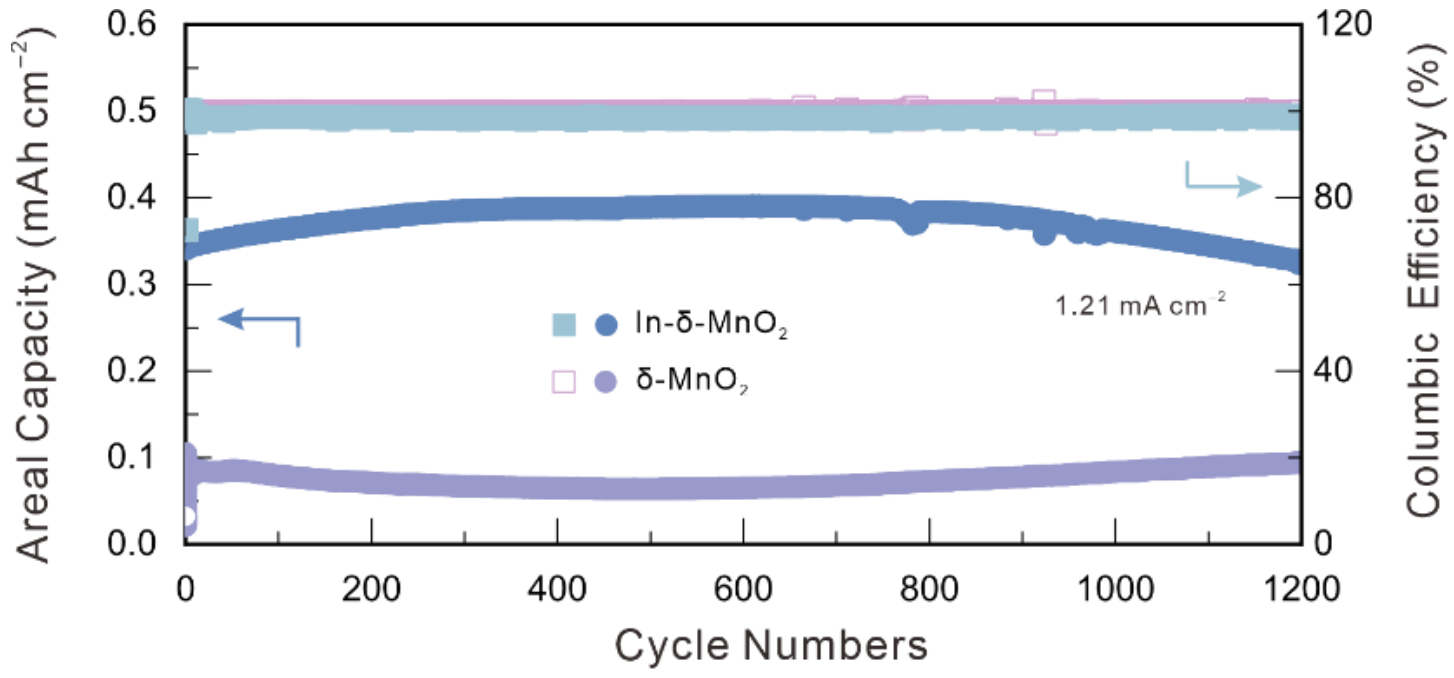

Figure S11. The GCD curves of In-δ-MnO_2 at different cycles.

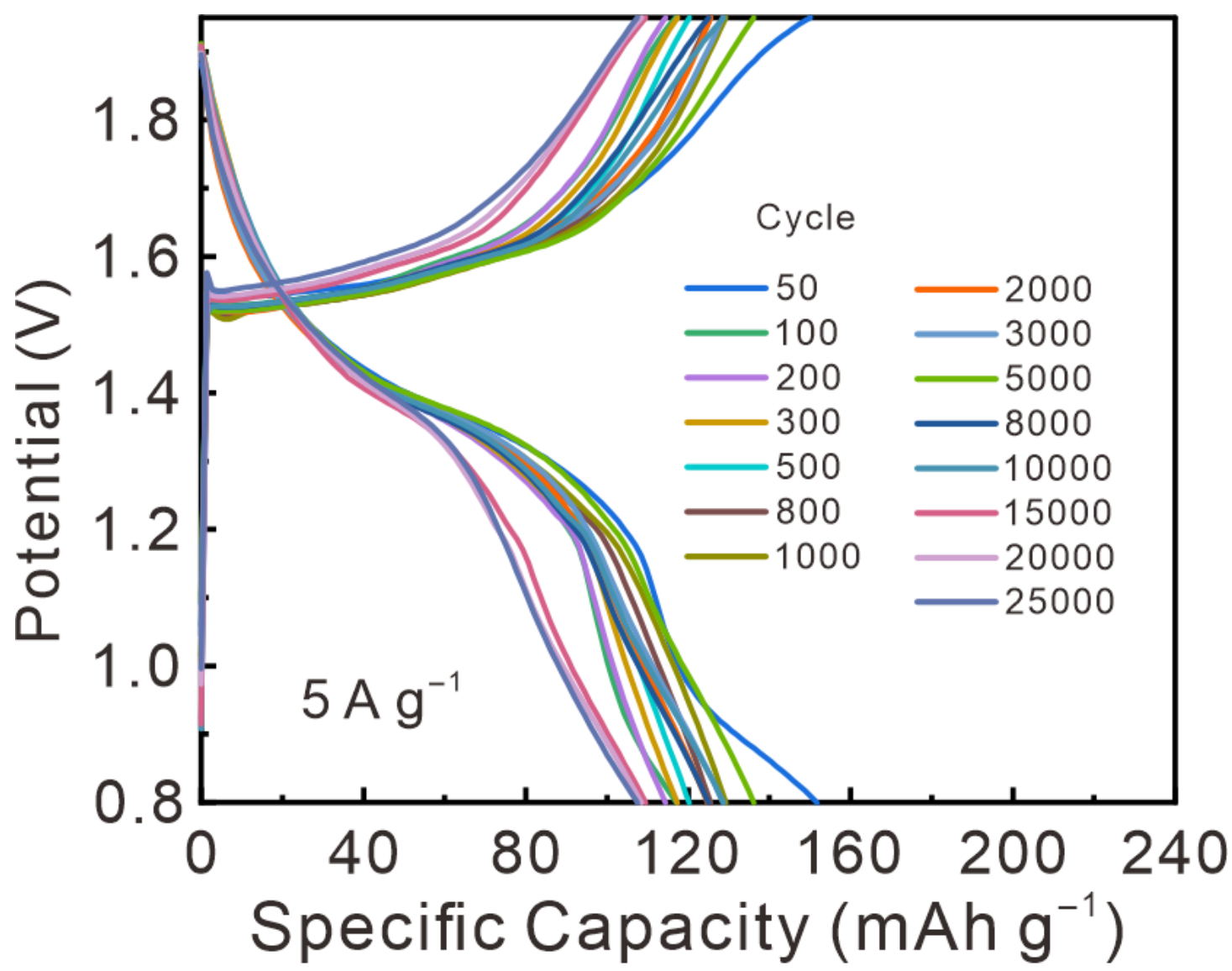

Figure S12. Cycling performance of In δ MnO_2 electrodes with various mass loadings (5.49-11.57 mg cm^{-2}) at 0.1 A g^{-1} for 50 cycles.

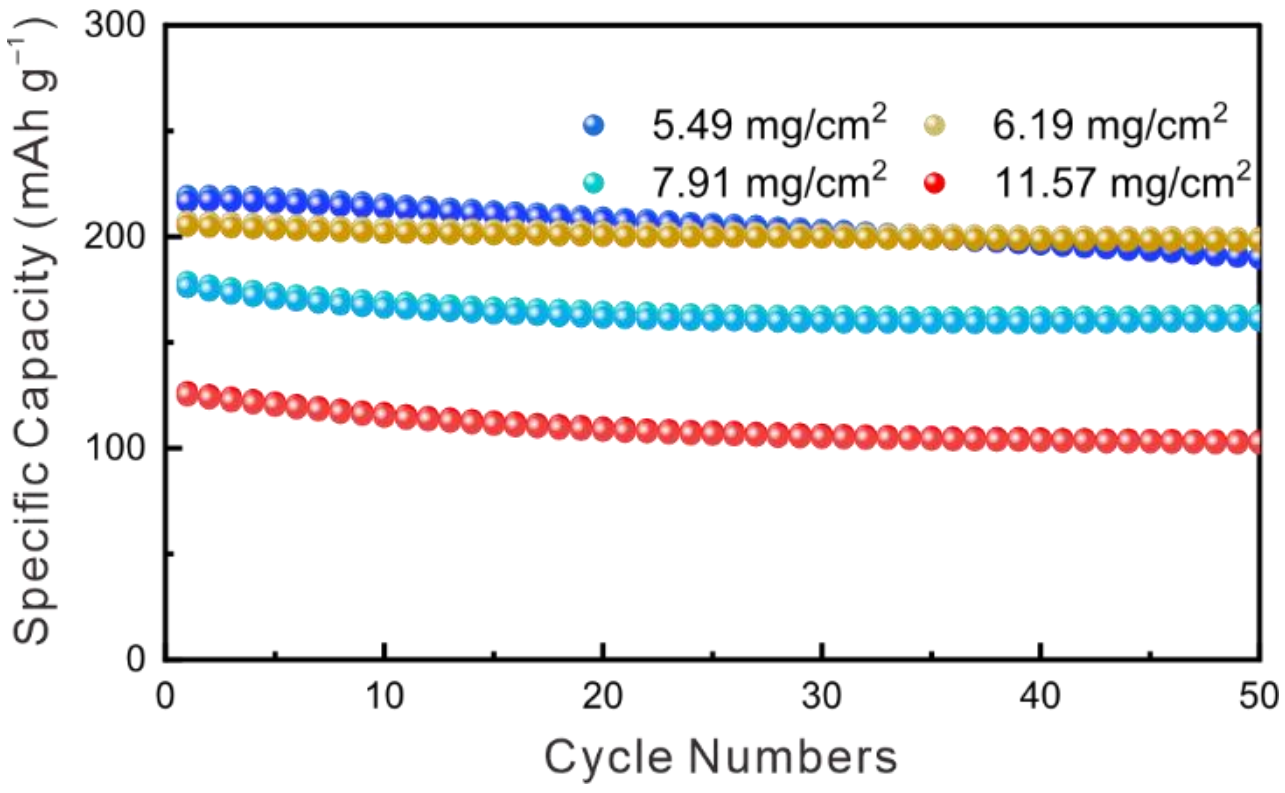

Figure S13. Cycling performance of In δ MnO_2 electrodes with a low N/P ratio at 0.1 A g^{-1} for 50 cycles.

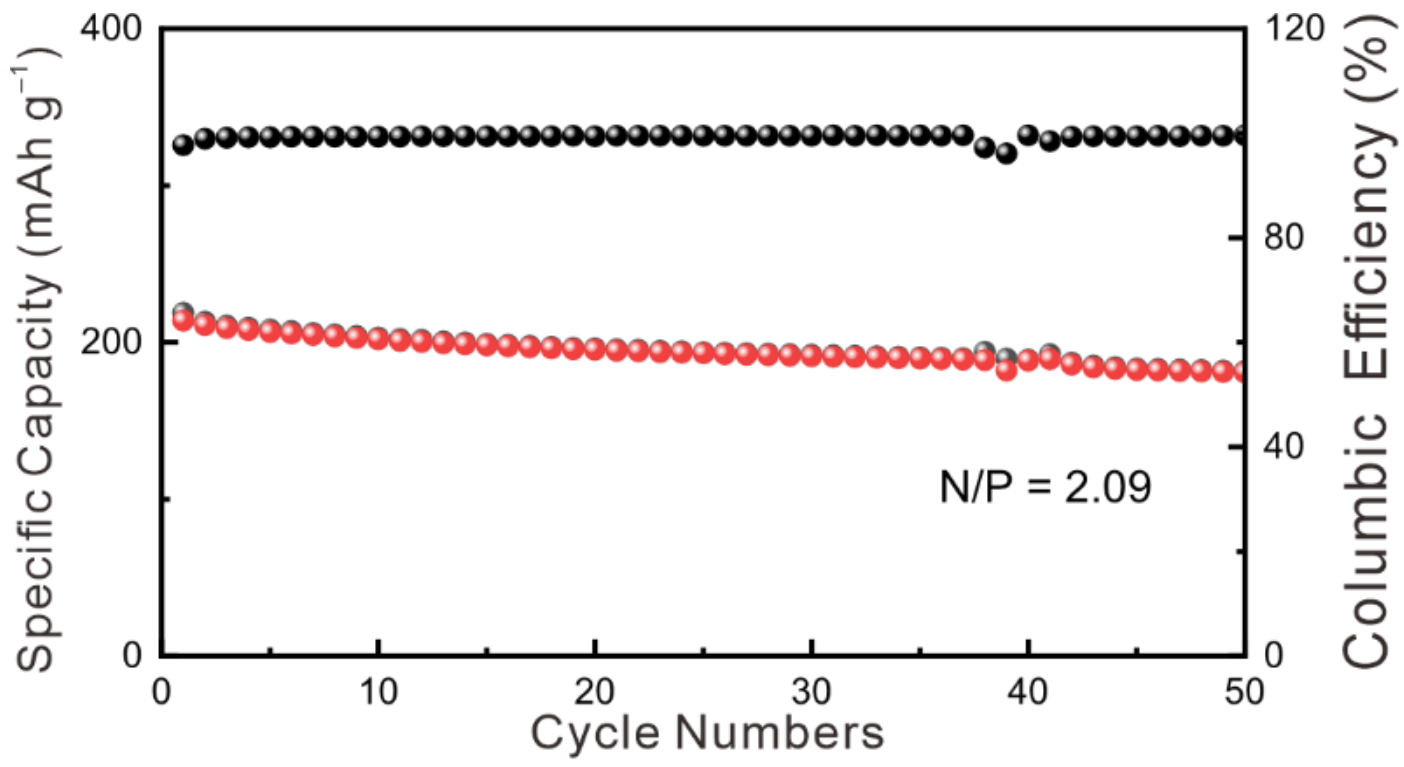

Figure S14. Cycling performance of In δ MnO_2 electrodes with a low electrolyte-to-capacity ratio at 0.1 A g^{-1} for 100 cycles.

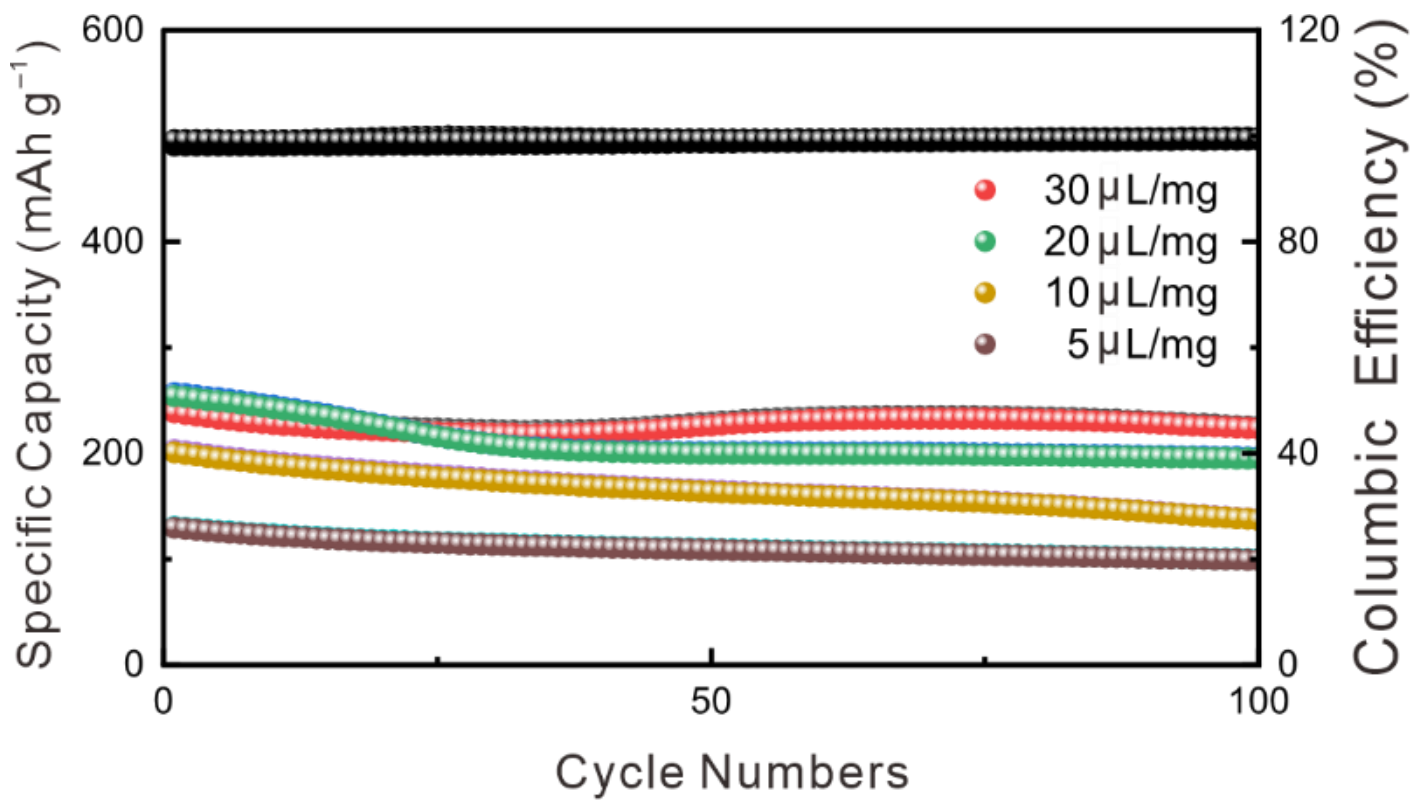

Figure S15. Schematic configuration of the assembled flexible pouch cell.

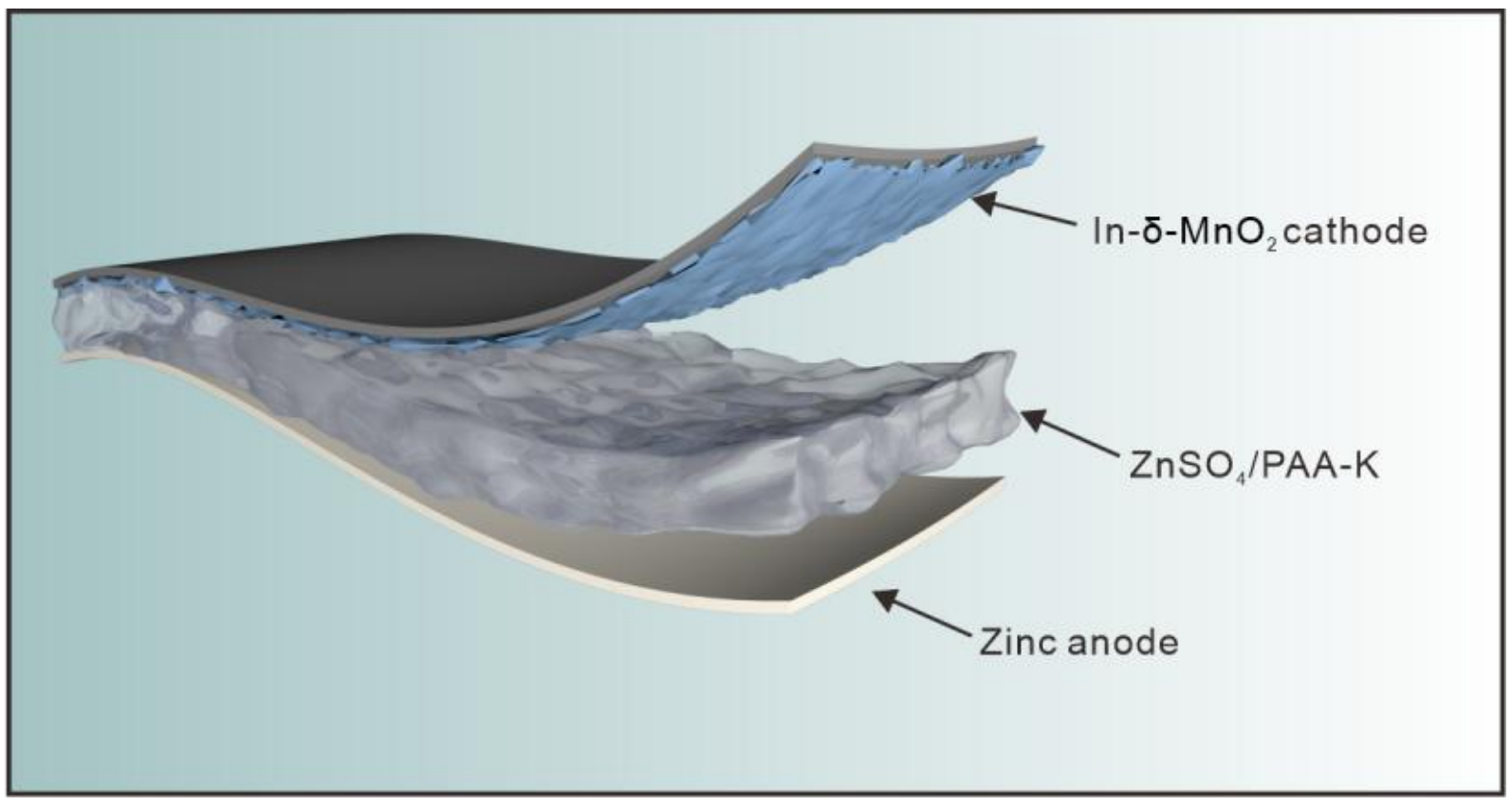

Figure S16. Structural illustration of the synthesized starch-reinforced polyacrylic acid hydrogel electrolyte.

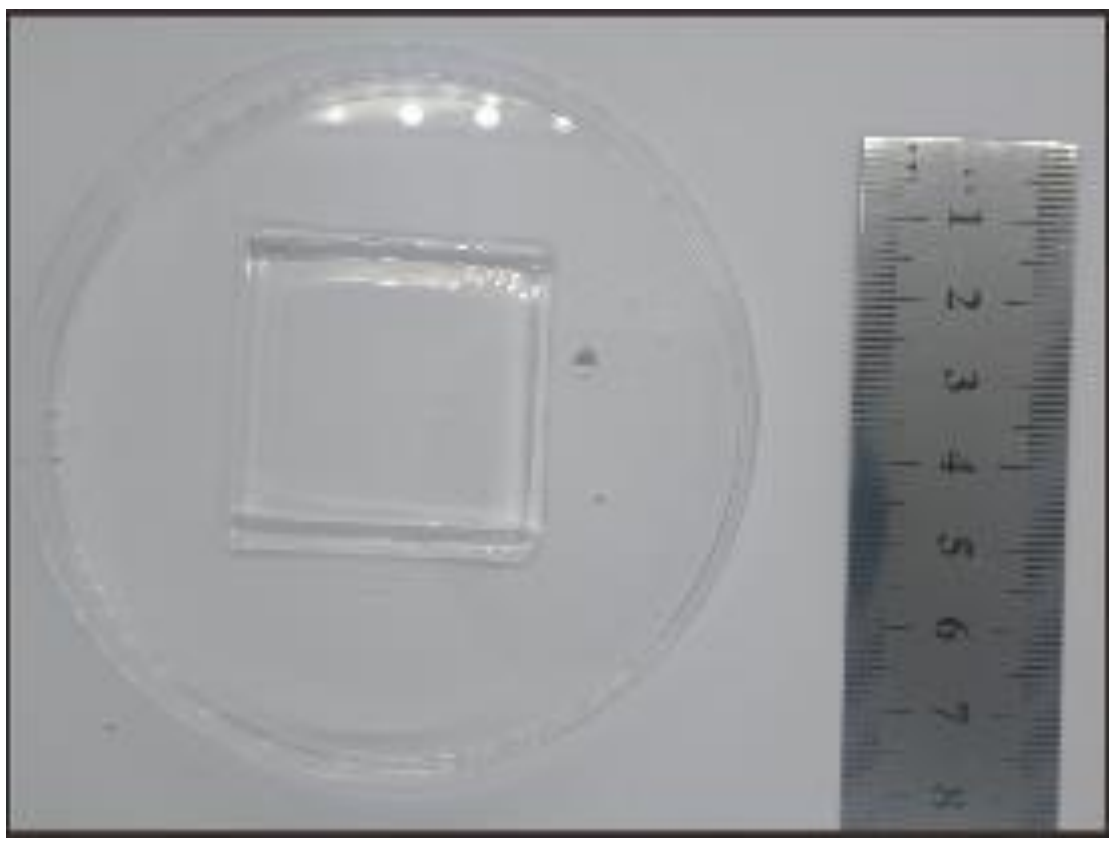

Figure S17. Bending diagram of hydrogel electrolyte.

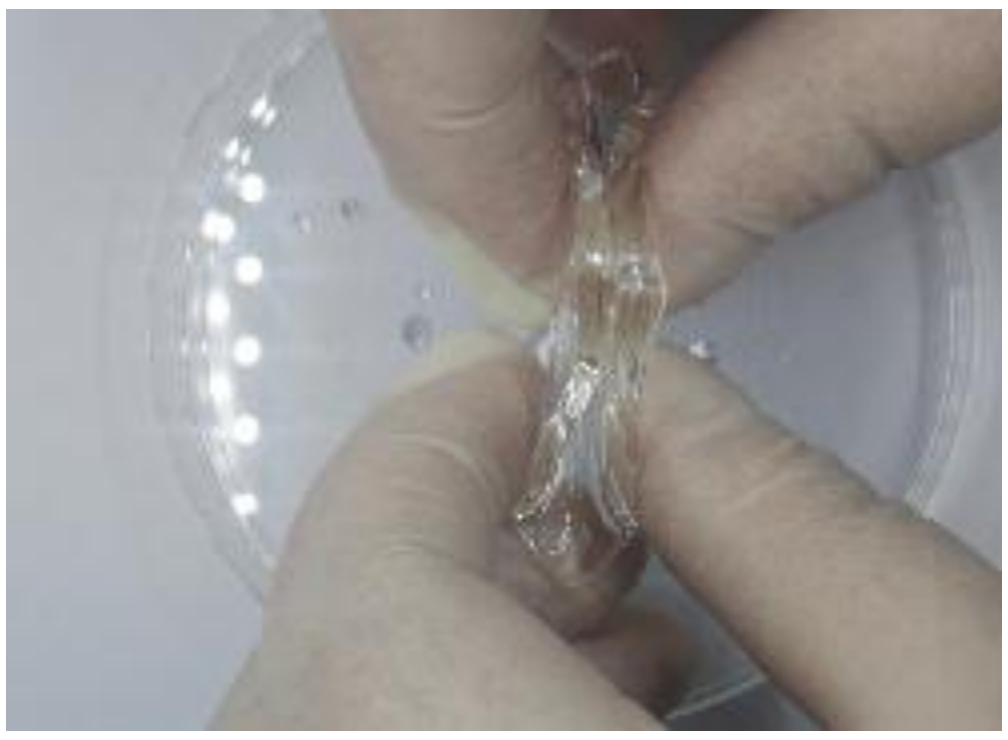

Figure S18. Optical image of a functioning flexible battery powering a commercial red LED.

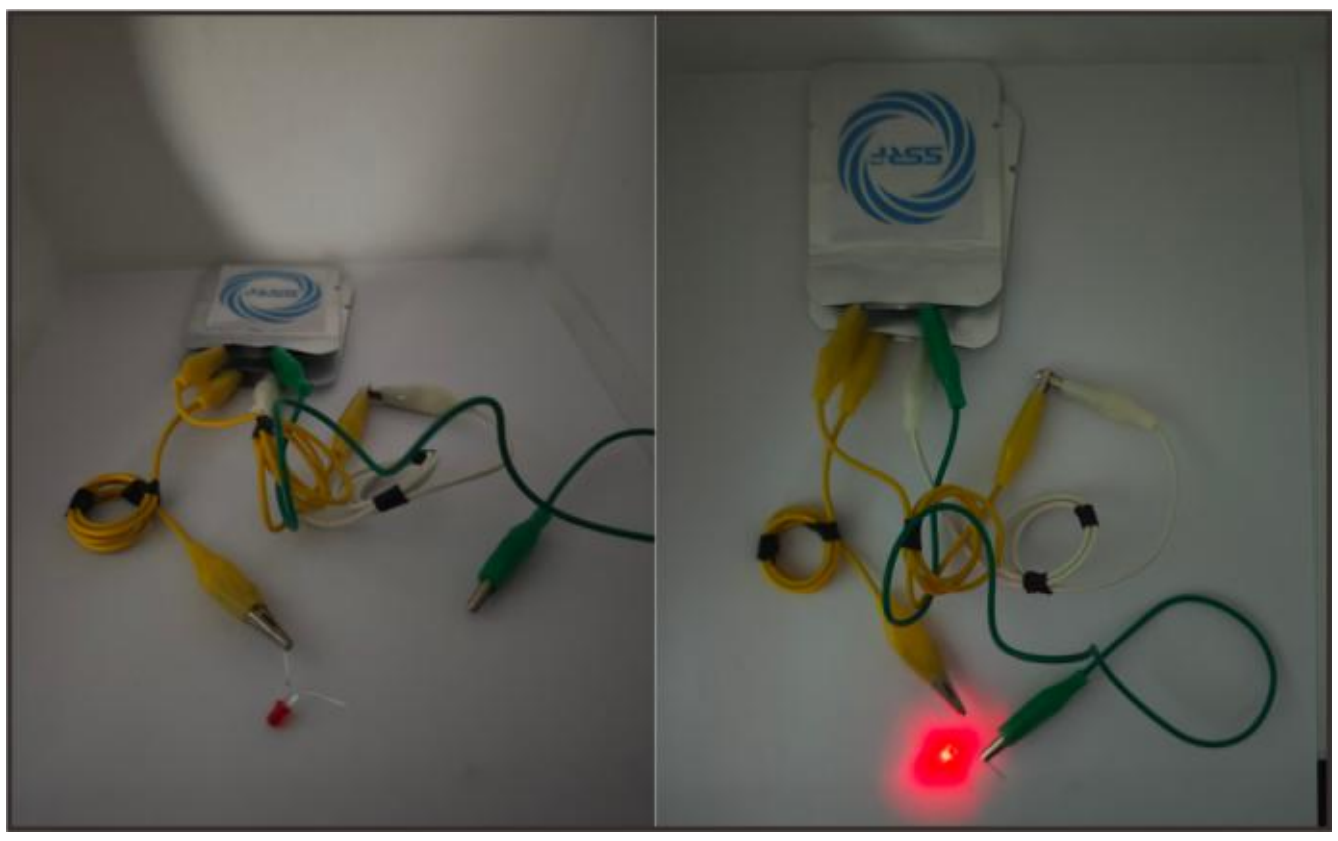

Figure S19. Cycling performance over 100 cycles at 0.2 A g^{-1}.

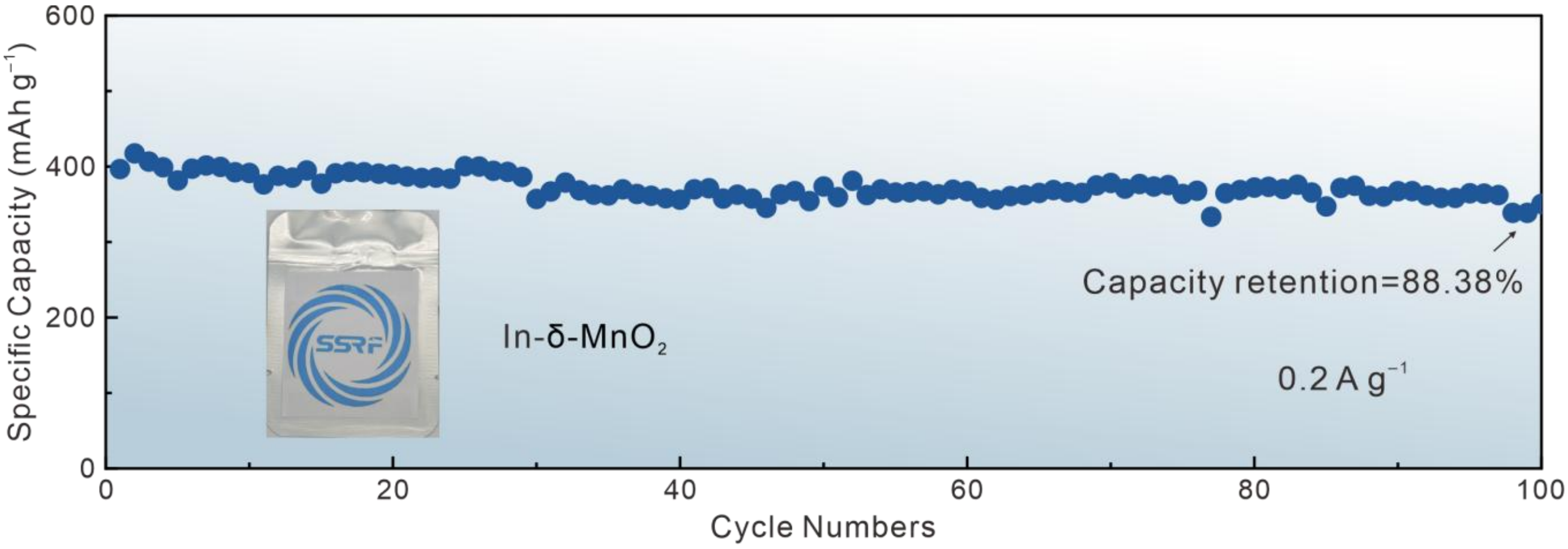

Figure S20. Galvanostatic charge-discharge profiles at selected cycles.

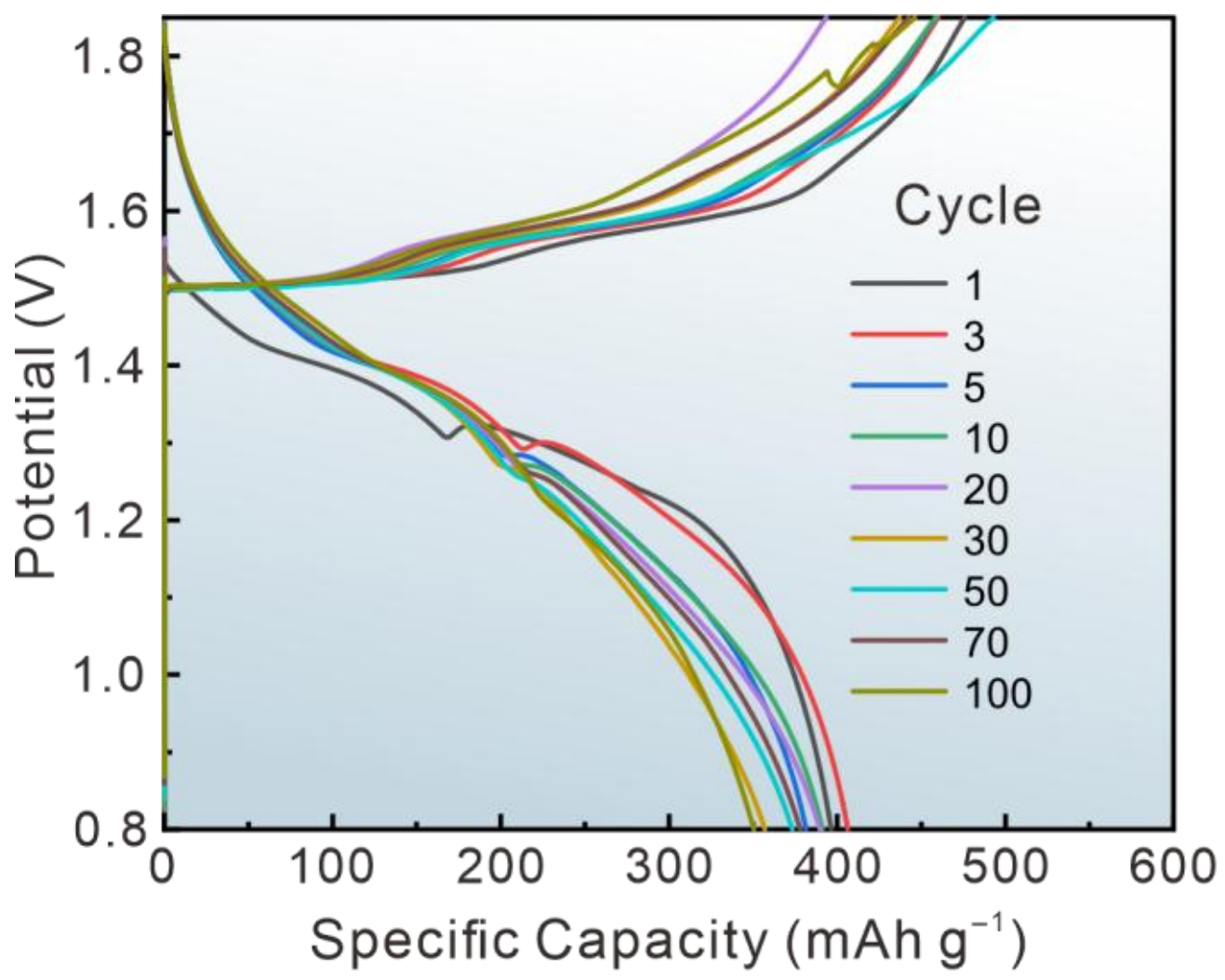

Figure S21. (a-d) Open-circuit voltage measurements under various bending conditions (0°, 90°, 135°, and 180°), demonstrating exceptional mechanical-electrochemical stability.

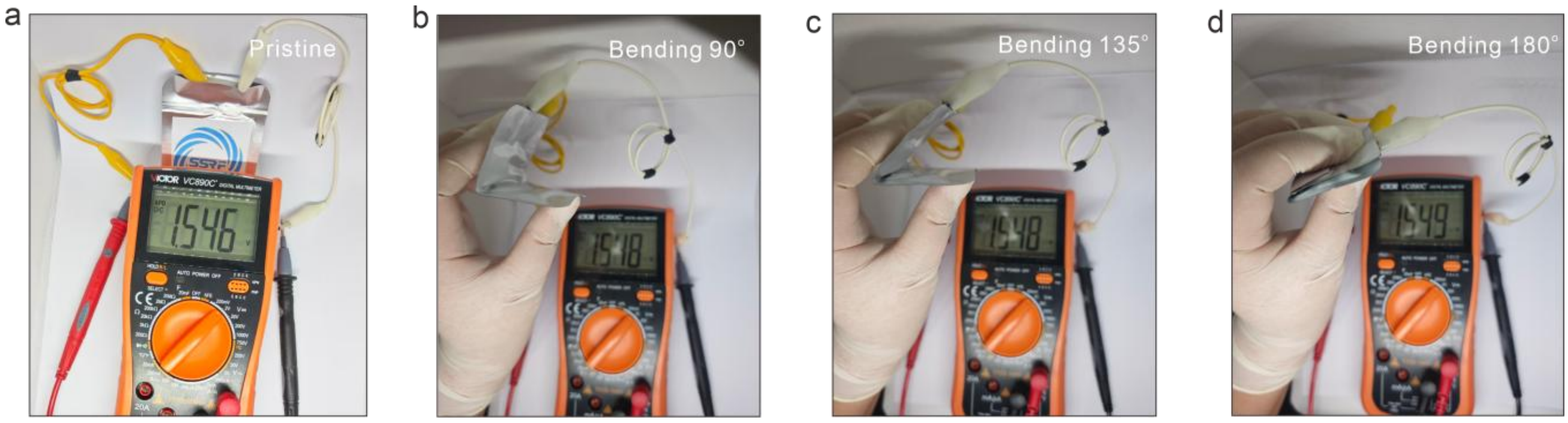

Figure S22. Schematic diagram of adsorption energy site structure.

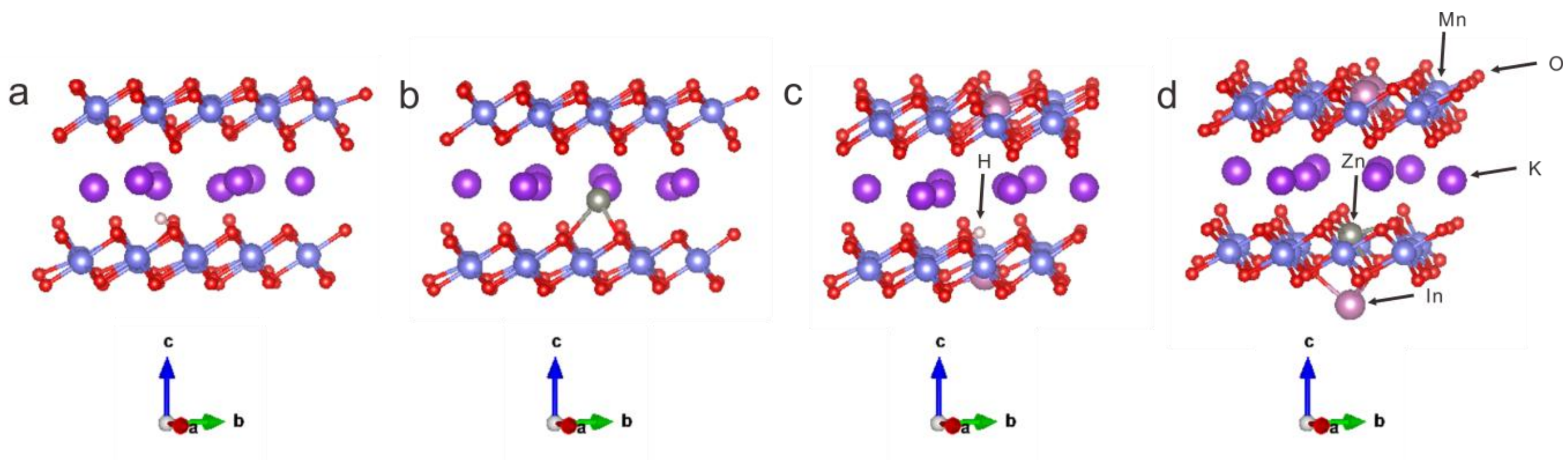

Figure S23. Migration structural schematic diagram.

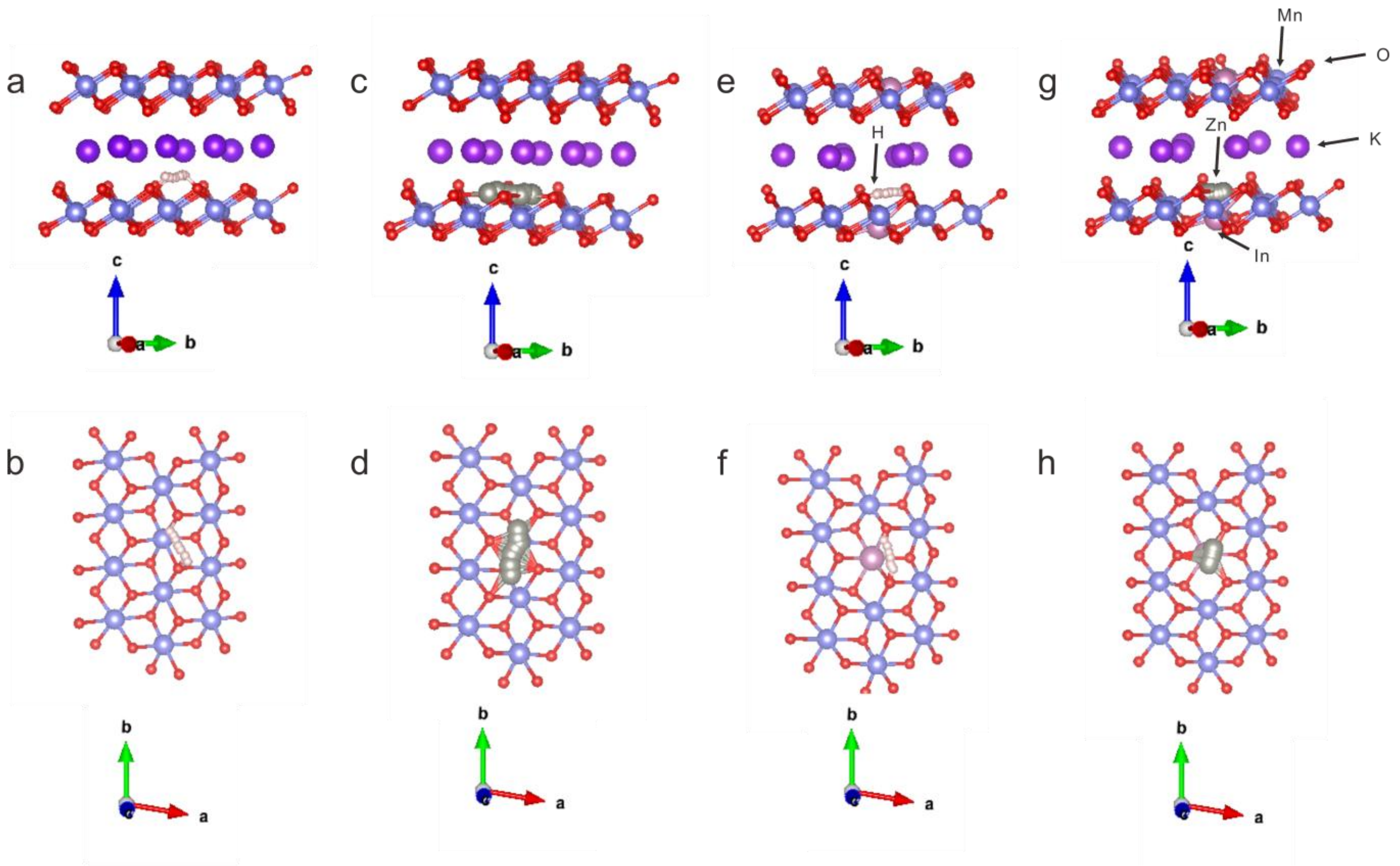

Element	wt%
**Mn**	41.89
**In**	11.41

Table S1. The mass percentages of Mn and In in In-δ-MnO_2 obtained by ICP-AES.

Sample	Conductivity (S/cm)
**δ-MnO_2**	0.00014468
**In-δ-MnO_2**	0.00022335

Table S2. Four-point probe conductivity measurements of δ-MnO_2 and In-δ-MnO_2.

Sample	Specific Current (A g^{-1})	Cycle Numbers	Specific capacity (mA g^{-1})	Year
MnO_2/C[6]	2	2000	~275	2026
MnO_2/AC[7]	1	1000	100.57	2026
Fe-MnO_2[8]	20	30000	110	2026
NiCoPBA@MnO_2[9]	1	800	86.2	2026
NG/MnO_2[10]	3	1000	68.6	2026
Ce-MnO_2[11]	0.6	1000	~120	2026
B-MnO_2[12]	3	2000	85	2026
CoMn-LDH@MnO_2/CC[13]	0.1	100	199.24	2026
Sn-MnO_2[14]	0.994	150	~600	2026
δ-MnO2/rGO[15]	1	500	172.73	2026
K-MnO_2[16]	2	2500	104	2026
$Mn_{1-x}V_xO_2$[17]	3	5000	113.6	2026
MC-MnO_2[18]	1	1000	~175	2025
δ-PMO[19]	1	1000	215	2025
CeCu- MnO_2[20]	0.5	2000	40	2025
AMC-MnO_2[21]	5	3000	~42	2025
α/R-MnO_2[22]	1	1000	~108	2025
P/C-MnO_2[23]	0.8	700	~107	2025
RC- MnO_2[24]	3	10000	141.61	2025
This work	5	25000	107.59	

Table S3. Comparison of the electrochemical performance between recently reported doped MnO_2 cathodes and the In-δ-MnO_2 cathode developed in this work.

Notes and references

[1] G. Kresse, J. Furthmüller, *Phys. Rev. B* **1996**, *54*, 11169-11186.

[2] J. P. Perdew, K. Burke, M. Ernzerhof, *Phys. Rev. Lett.* **1996**, *77*, 3865-3868.

[3] J. J. Ye, P. H. Li, Z. Hou, W. Zhang, W. Zhu, S. Jin, H. Ji, *Angew. Chem. Int. Ed.* **2024**, *63*, e202410900.

[4] S. Grimme, J. Antony, S. Ehrlich, H. Krieg, *J. Chem. Phys.* **2010**, *132*, 154104.

[5] X. Zhao, F. Zhang, H. Li, H. Dong, C. Yan, C. Meng, Y. Sang, H. Liu, Y. G. Guo, S. Wang, *Energy Environ. Sci.* **2024**, *17*, 3629-3640.

[6] H. Yuan, S. Luo, Y. Guo, B. Yuan, H. Gao, K. Wang, W. Yang, L. Xu, Y. Luo, G. Yang, Y. Wang, *J. Energy Storage* **2026**, *161*, 122005.

[7] S. Jiang, M. Xu, X. Wang, S. Zhang, C. Zhao, S. Tian, X. Xue, *J. Energy Storage* **2026**, *160*, 122023.

[8] H. Hu, Y. Feng, Z. Wang, L. Zhuo, C. Hu, I. Shakir, D. Wang, X. Liu, *Energy Environ. Sci.* **2026**, *19*, 1603-1615.

[9] H. Sun, L. Sun, W. Li, J. Li, Y. Wang, J. Cui, *J. Mater. Chem. A* **2026**, *14*, 14957-14968.

[10] X. Wu, J. Zhang, G. Zhang, Y. Cao, X. Hua, Y. Xu, J. Zhang, Q. Jiang, H. Yang, Y. Luo, X. Song, J. Wang, W. Li, X. Li, *ACS Appl. Energy Mater.* **2026**, *9*, 2992-3004.

[11] Y. Zhu, X. Wang, J. Xia, X. Wang, Y. Kong, Y. Zhou, S. Qu, W. Feng, J. Di, *Nanoscale* **2026**, 18, 6321-6328.

[12] D. Zou, F. Liu, S. Y. Zhou, Y. S. Ran, Y. D. Zheng, H. Wen, X. D. Wang, C. L. Yang, *J. Alloys Compd.* **2026**, 1053.

[13] B. Ma, Y. Zhang, S. Wang, F. Li, Y. Wang, W. Zhang, *Electrochim. Acta* **2026**, *546*, 147811.

[14] J. Tong, Y. Chen, Z. Wei, J. Liu, Y. Ge, Q. Tong, Y. Cheng, Z. Yu, *J. Alloys Compd.* **2026**, *1050*, 185517.

[15] C. Liu, Q. Gou, Y. Chen, Y. Tian, H. Mei, M. Qiao, *J. Mater. Chem. A* **2026**, *14*, 4501-4510.

[16] J. X. Yang, Z. Yu, F. Zhang, J. J. Zheng, J. Zhao, X. Y. Wang, *J. Energy Storage* **2026**, 143.

[17] X. Zhang, X. Yang, Y. Wei, D. Wang, Y. Chen, *J. Mater. Chem. A* **2026**, *14*, 1183-1194.

[18] Z. Li, L. Mao, C. Yin, C. Wu, *Chem. Eng. J.* **2025**, *526*, 171200.

[19] K. Li, H. Zhang, J. Wang, Y. Liang, T. Liu, S. Guo, Z. Jin, Z. Zhang, C. Guo, B. Cong, B. Wang, *Chem. Eng. J.* **2026**, *528*, 172351.

[20] Y. Pang, B. Jiao, J. Wang, Y. Wang, Z. Li, B. Zhao, Y. Pan, *ACS Appl. Energy Mater.* **2025**, *8*, 16641-16648.

[21] Y. Wang, H. Zhou, S. Wei, S. Chen, D. Cao, Q. Zhou, X. Wu, L. Song, *J. Am. Chem. Soc.* **2025**, 147, 41297-41307.

[22] S. Wang, K. Ji, J. Yang, S. Yao, J. Wu, Y. Ji, W. Bai, R. Liu, X. Li, W. Ren, G. Wang, J. Wang, F. Zhang, Z. Yang, Y.-M. Yan, *Nano Energy* **2025**, *146*, 111561.

[23] H. K. Wen, R. Y. Ge, Y. W. Shi, S. W. Zhang, H. Y. Wen, H. Fan, H. Li, B. S. Yin, T. Y. Ma, *J. Energy Storage* **2025**, 140.

[24] Z. Li, C. Wu, C. Yin, *Electrochim. Acta* **2025**, *542*, 147537.
